# Supplementary material for: Stromal heterogeneity may explain increased incidence of metaplastic breast cancer in women of African descent
Source: Nat Commun. 2023 Sep 14;14:5683. doi: 10.1038/s41467-023-41473-6 (PMC10502140; doi:10.1038/s41467-023-41473-6)
Supplement: Supplementary file 1 — Supplementary Information [file 41467_2023_41473_MOESM1_ESM.pdf]

Stromal heterogeneity may explain increased incidence of metaplastic breast cancer in women of African descent.

Brijesh Kumar[1,8,9], Aditi S. Khatpe[1,2,8], Jiang Guanglong[3], Katie Batic[1], Poornima Bhat-Nakshatri[1], Maggie M. Granatir[4], Rebekah Joann Addison[4], Megan Szymanski[4], Lee Ann Baldridge[4], Constance J. Temm[4], George Sandusky[4], Sandra K. Althouse[5], Michele L. Cote[6], Kathy D. Miller[3], Anna Maria Storniolo[3], and Harikrishna Nakshatri[1,2,7,10]

[1]Department of Surgery, Indiana University School of Medicine, Indianapolis, IN 46202, USA

[2]Department of Biochemistry and Molecular Biology, Indiana University School of Medicine, Indianapolis, IN 46202, USA.

[3]Department of Medicine, Indiana University School of Medicine, Indianapolis, IN 46202, USA

[4]Department of Pathology and Laboratory Medicine, Indiana University School of Medicine, Indianapolis, IN 46202, USA

[5]Department of Biostatistics and Health Data Science, Indiana University School of Medicine, Indianapolis, IN 46202, USA

[6]Richard M. Fairbanks School of Public Health, Indiana University, Indianapolis, IN 46202, USA

[7]VA Roudebush Medical Center, Indianapolis, IN 46202, USA

[8]These authors contributed equally.

[9]Current Address: School of Biomedical Engineering, Indian Institute of Technology (Banaras Hindu University), Varanasi, UP 221005, India

[10]Corresponding author: Harikrishna Nakshatri, B.V.Sc., PhD.

C218C, 980 West Walnut Street

Indianapolis, IN 46202, USA

317 278 2238 (phone)

317 274 0396 (fax)

email: hnakshat@iupui.edu

Running title: Influence of genetic ancestry on breast stromal cells.

Keywords: breast cancer, genetic ancestry, normal breast, PROCR, ZEB1, PDGFRα, IL-6, STAT3, breast microenvironment, metaplastic carcinoma

Supplementary Figures and Tables.

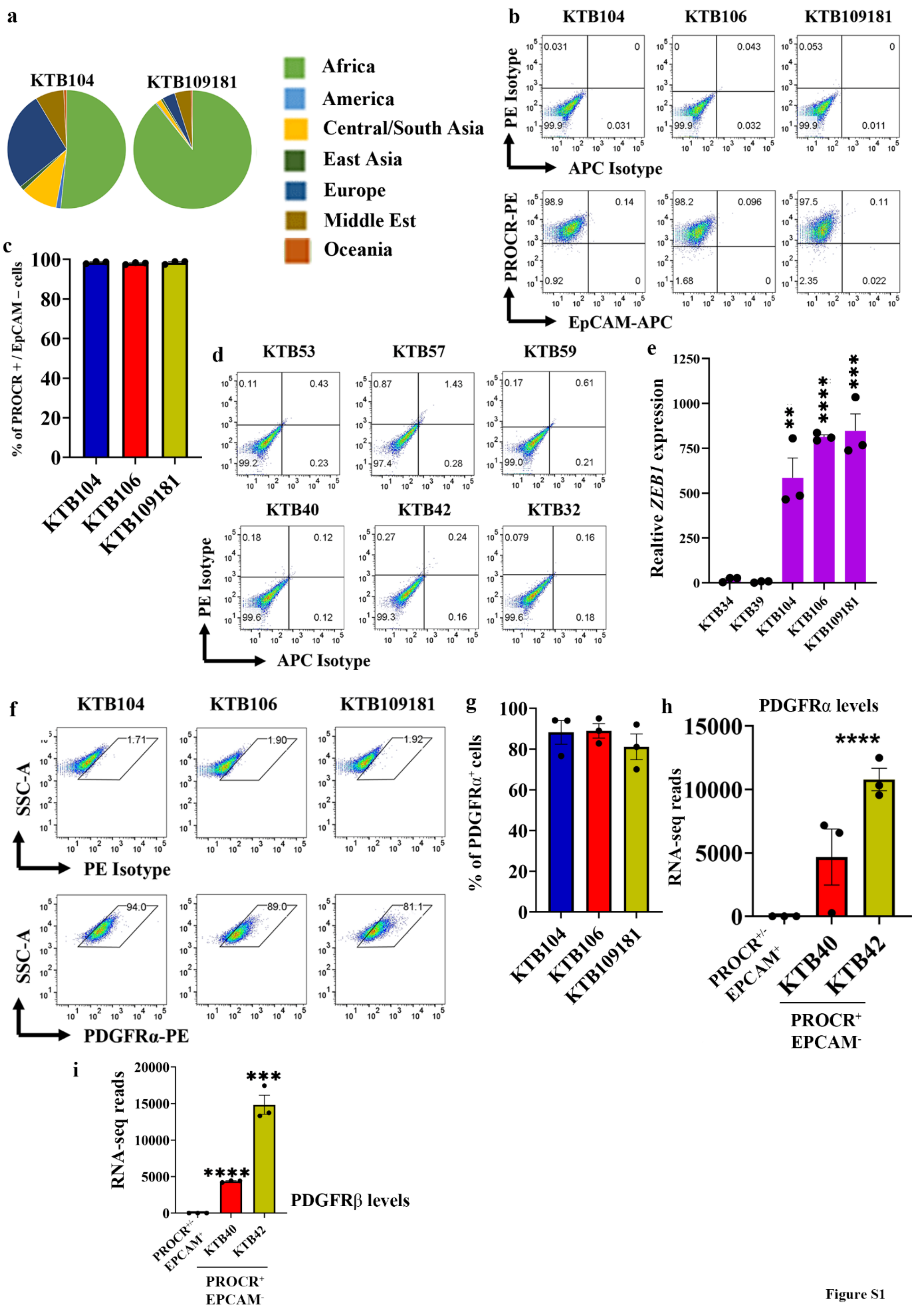

a
KTB104
KTB109181
Africa
America
Central/South Asia
East Asia
Europe
Middle Est
Oceania
b
KTB104
KTB106
KTB109181
PE Isotype
APC Isotype
PROCR-PE
EpCAM-APC
c
% of PROCR + / EpCAM – cells
KTB104
KTB106
KTB109181
d
KTB53
KTB57
KTB59
KTB40
KTB42
KTB32
PE Isotype
APC Isotype
e
Realtive ZEB1 expression
KTB34
KTB39
KTB104
KTB106
KTB109181
f
KTB104
KTB106
KTB109181
SSC-A
PE Isotype
SSC-A
PDGFRα-PE
g
% of PDGFRα+ cells
KTB104
KTB106
KTB109181
h
PDGFRα levels
RNA-seq reads
PROCR+/- EPCAM+
KTB40
KTB42
PROCR+ EPCAM-
i
RNA-seq reads
PDGFRβ levels
PROCR+/- EPCAM+
KTB40
KTB42
PROCR+ EPCAM-

Figure S1

Figure S1: Establishment of PROCR$^+$/ZEB1$^+$/PDGFRα$^+$ (PZP) cell lines from the healthy breast tissues of women of African ancestry. (a) Genetic ancestry mapping of breast tissue donors (KTB104, and KTB109181) using a 41-SNP panel. (b) PROCR$^+$/EpCAM$^–$ cells are enriched in established PZP cell lines (n=3). (c) Quantitation of PROCR$^+$/EpCAM$^–$ cells (n=3). (d) APC and PE isotypes control staining patterns of cell lines, as characterized by flow cytometry (n=3). These staining patterns were used to draw quadrants. (E) ZEB1 expression levels in various PROCR$^+$/EpCAM$^–$ (KTB104*p=0.0065, KTB106*p<0.0001 and KTB109181*p=0.0009) cell lines compared to EpCAM$^+$ (KTB34 and KTB39) luminal cell lines (n=3). Data analyzed using two-tailed t-test. (f) PROCR$^+$/ZEB1$^+$ (KTB104, KTB106 and KTB109181) cell lines express PDGFRα as determined by flow cytometry (n=3). (g) Quantitation of PDGFRα$^+$ cells (n=3). (h) PDGFRα mRNA level in PROCR$^+$/EpCAM$^–$ cells compared to PROCR$^±$/EpCAM$^+$ cells. These results are from RNA-seq studies done in triplicate that involved seven epithelial cell lines, KTB40 and KTB42 (*p=0.0003 and results have been published previously [1]). Data analyzed using one-way ANOVA. (i) PDGFRβ mRNA level in PROCR$^+$/EpCAM$^-$ cells compared to PROCR$^±$/EpCAM$^+$ cells (KTB*p<0.0001; KTB42*p=0.0004). Data analyzed using two tailed t-test. **p<0.01, ***p<0.001, ****p<0.0001. All the data points are shown as mean ± SEM. Source data are provided as a Source Data file.

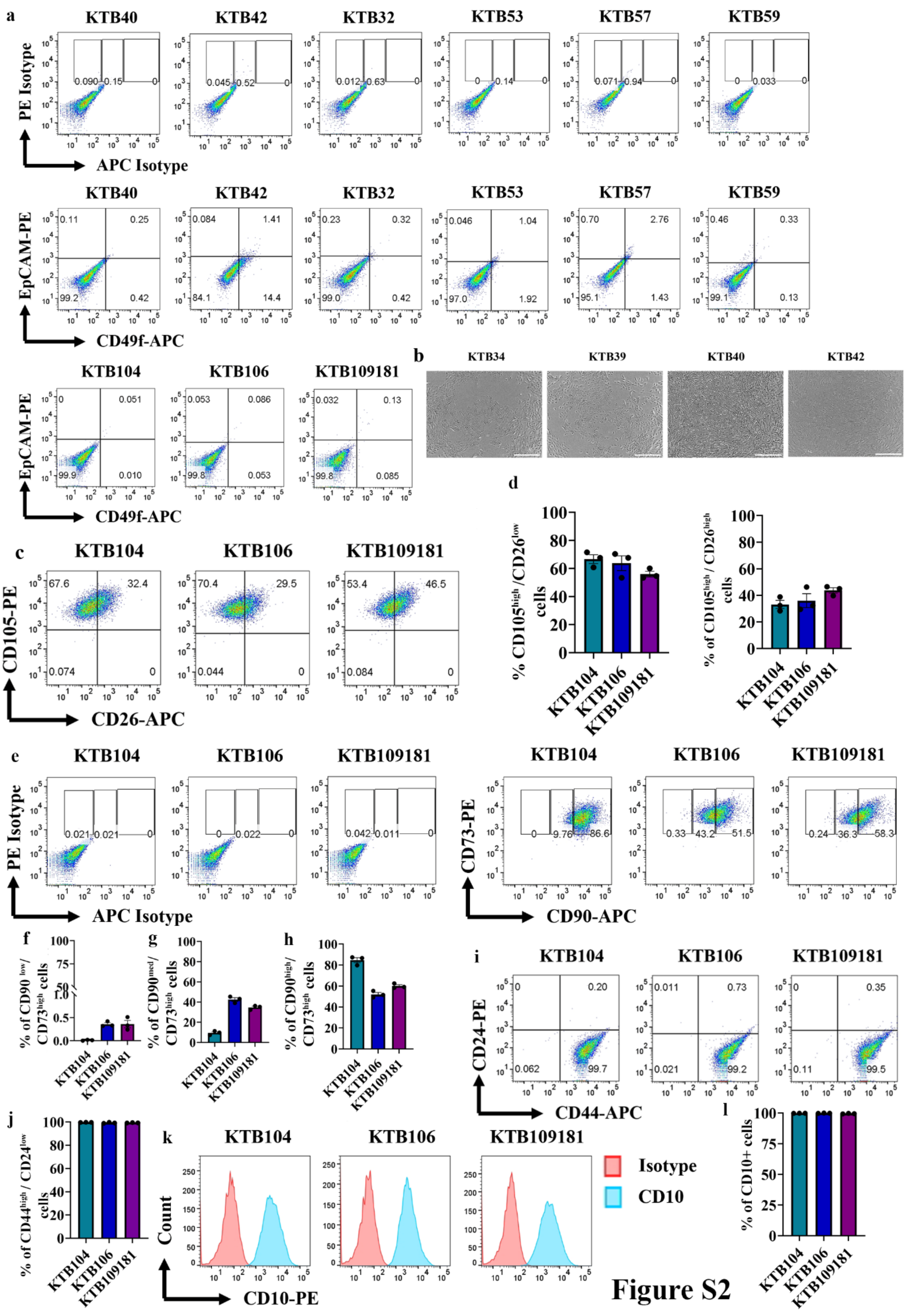

a
KTB40
KTB42
KTB32
KTB53
KTB57
KTB59
PE Isotype
APC Isotype
EpCAM-PE
CD49f-APC
KTB104
KTB106
KTB109181
b
KTB34
KTB39
KTB40
KTB42
c
CD105-PE
CD26-APC
d
% CD105high /CD26low cells
% of CD105high / CD26high cells
e
CD73-PE
CD90-APC
f
% of CD90 low/ CD73high cells
g
% of CD90med/ CD73high cells
h
% of CD90high/ CD73high cells
i
CD24-PE
CD44-APC
j
% of CD44high / CD24low cells
k
Count
CD10-PE
Isotype
CD10
l
% of CD10+ cells

Figure S2

Figure S2: Additional cell surface marker profiles of PROCR^{+}/ZEB1^{+} cells. (a) APC and PE isotypes control staining patterns of cell lines as characterized by flow cytometry (n=3). These staining patterns were used to draw quadrants. PZP cell lines were stained with CD49f and EpCAM antibodies to demonstrate lack of breast basal, luminal progenitor, and mature/differentiated cell population (n=3). (b) Phase contrast images representing the epithelial morphology of human telomerase gene (hTERT) immortalized breast epithelial cells (KTB34 and KTB39) and fibroblast-like features of KTB40 and KTB42 (n=3). (c) PZP cell lines were stained with CD105 and CD26 antibodies to determine whether PZP cells show phenotypic similarity to the lobular and interlobular human breast fibroblastic cells (n=3). (d) Quantification of CD105^{high}/CD26^{-} and CD105^{high}/CD26^{low} population of cells (n=3). (E) PZP cell lines were stained with CD90 and CD73 antibodies to identify rare endogenous pluripotent somatic stem cells and potential mesenchymal stem cells (n=3). (f, g and h) Quantification of CD90^{low} /CD73^{high}, CD90^{med}/CD73^{high}, and CD90^{high}/CD73^{high} population of cells (n=3). (i) PZP cell lines were stained with CD44 and CD24 antibodies to determine whether their phenotype overlaps with cancer stem cells (n=3). (j) Quantification of CD44^{high}/CD24^{low} population (n=3). (k) PZP cell lines were stained with CD10 antibody to determine myoepithelial cell marker expression (n=3). (l) Quantification of CD10^{+} population (n=3). All the data points are shown as mean ± SEM. Source data are provided as a Source Data file.

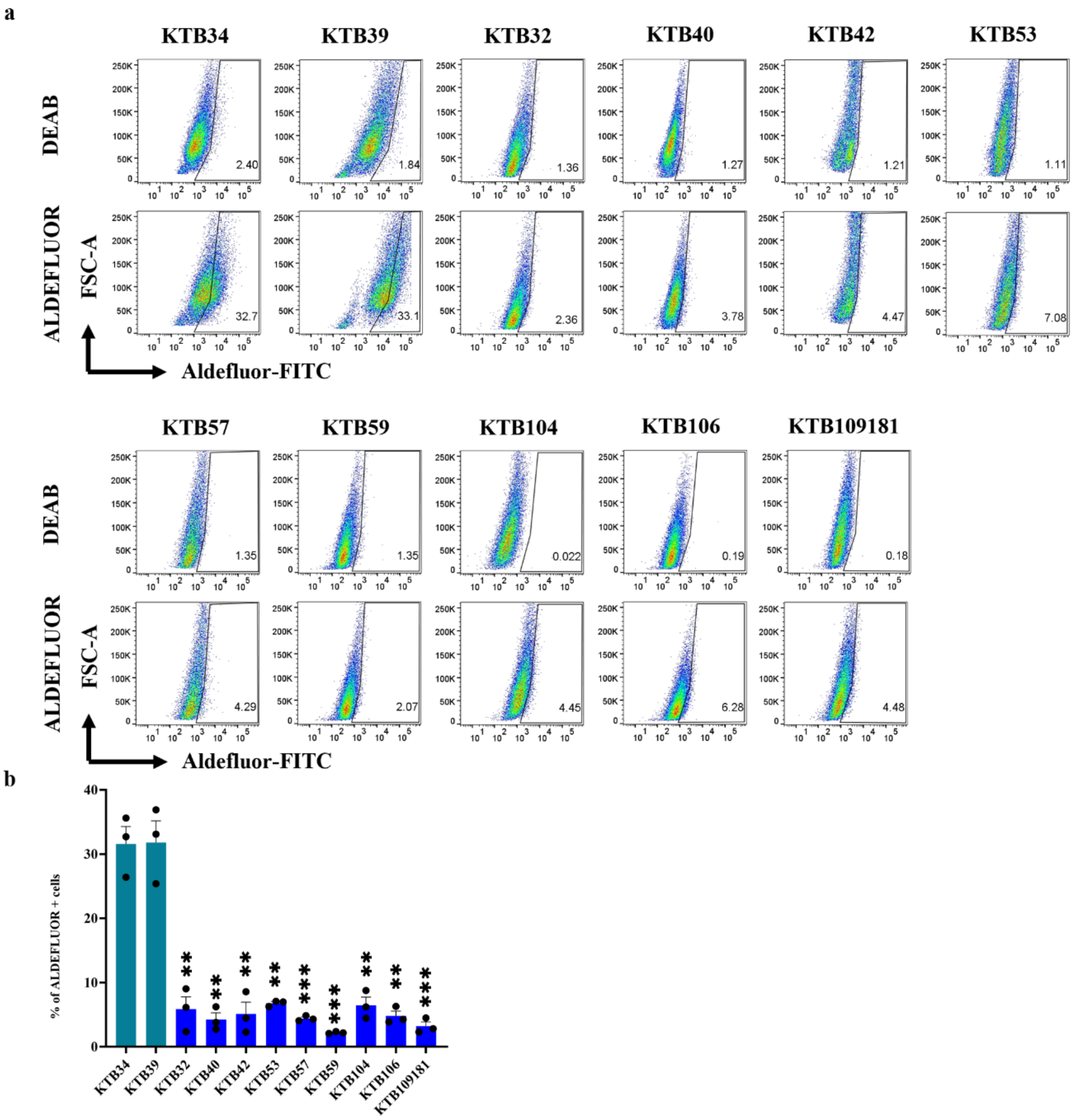
a
KTB34
KTB39
KTB32
KTB40
KTB42
KTB53
DEAB
ALDEFLUOR
FSC-A
Aldefluor-FITC
2.40
1.84
1.36
1.27
1.21
1.11
32.7
33.1
2.36
3.78
4.47
7.08
KTB57
KTB59
KTB104
KTB106
KTB109181
1.35
1.35
0.022
0.19
0.18
4.29
2.07
4.45
6.28
4.48
b
% of ALDEFLUOR + cells

Figure S3

Figure S3: PZP cells lack ALDH+ subpopulation. (a) ALDH+ subpopulation, as measured through ALDEFLUOR assay, in immortalized breast epithelial (KTB34 and KTB39) and PZP (KTB32, KTB40, KTB42, KTB53, KTB57, KTB59, KTB104, KTB106 and KTB109181) cell lines. (b) Quantification of ALDH+ cells (n=3). Data analyzed using two tailed t-test (KTB32*p=0.002, KTB40*p=0.001, KTB42*p=0.0017, KTB53*p=0.0012, KTB57*p=0.0009, KTB59*p=0.0006, KTB104*p=0.0016, KTB106*p=0.001 and KTB109181*p=0008 compared to epithelial cell lines). All the data points are shown as mean ± SEM. Source data are provided as a Source Data file.

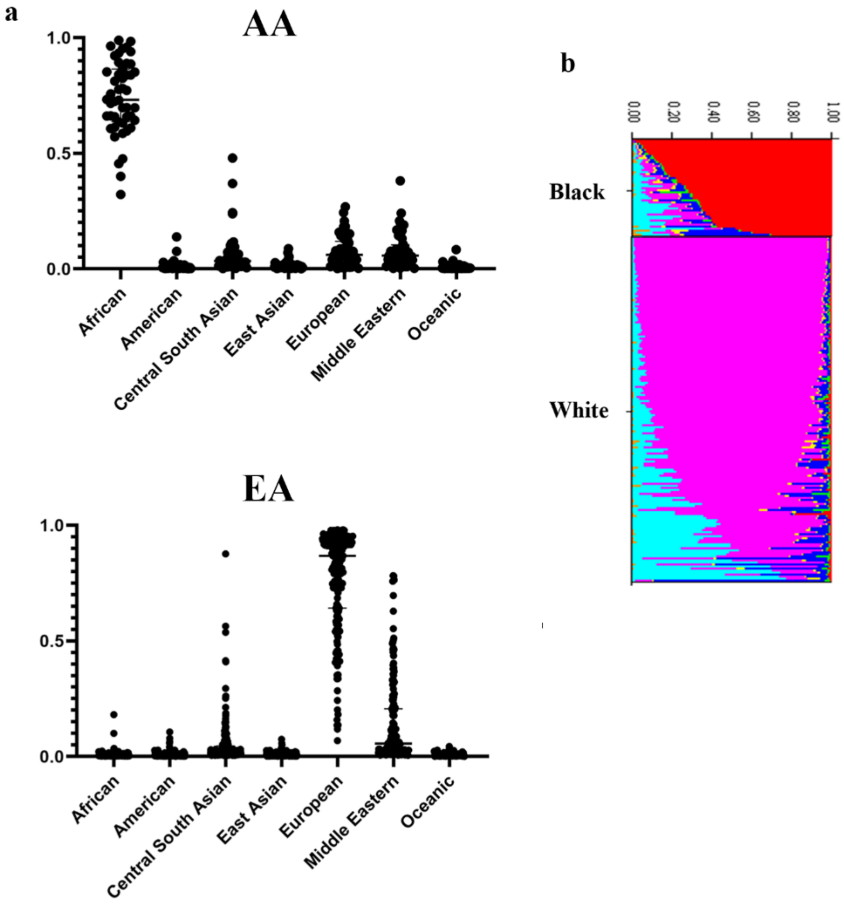
a
AA
1.0
0.5
0.0
African
American
Central South Asian
East Asian
European
Middle Eastern
Oceanic
EA
1.0
0.5
0.0
African
American
Central South Asian
East Asian
European
Middle Eastern
Oceanic
b
0.00
0.20
0.40
0.60
0.80
1.00
Black
White

Figure S4

Figure S4: Genetic ancestry mapping of African American and non-Hispanic White breast tissue donors. (a) Genetic ancestry marker distribution patterns of donors whose Normal- Healthy breast tissues were used to generate TMA. (b) The bar plot of population structure from STRUCTURE Bayesian analysis with K=7. Each individual is represented by a vertical bar with colors representing the estimated proportion of ancestry. The reference populations are Africa (red), America (green), Europe (magenta), Central/South Asia (blue), East Asia (yellow), Middle East (Cyan), and Oceania (orange). Source data are provided as a Source Data file.

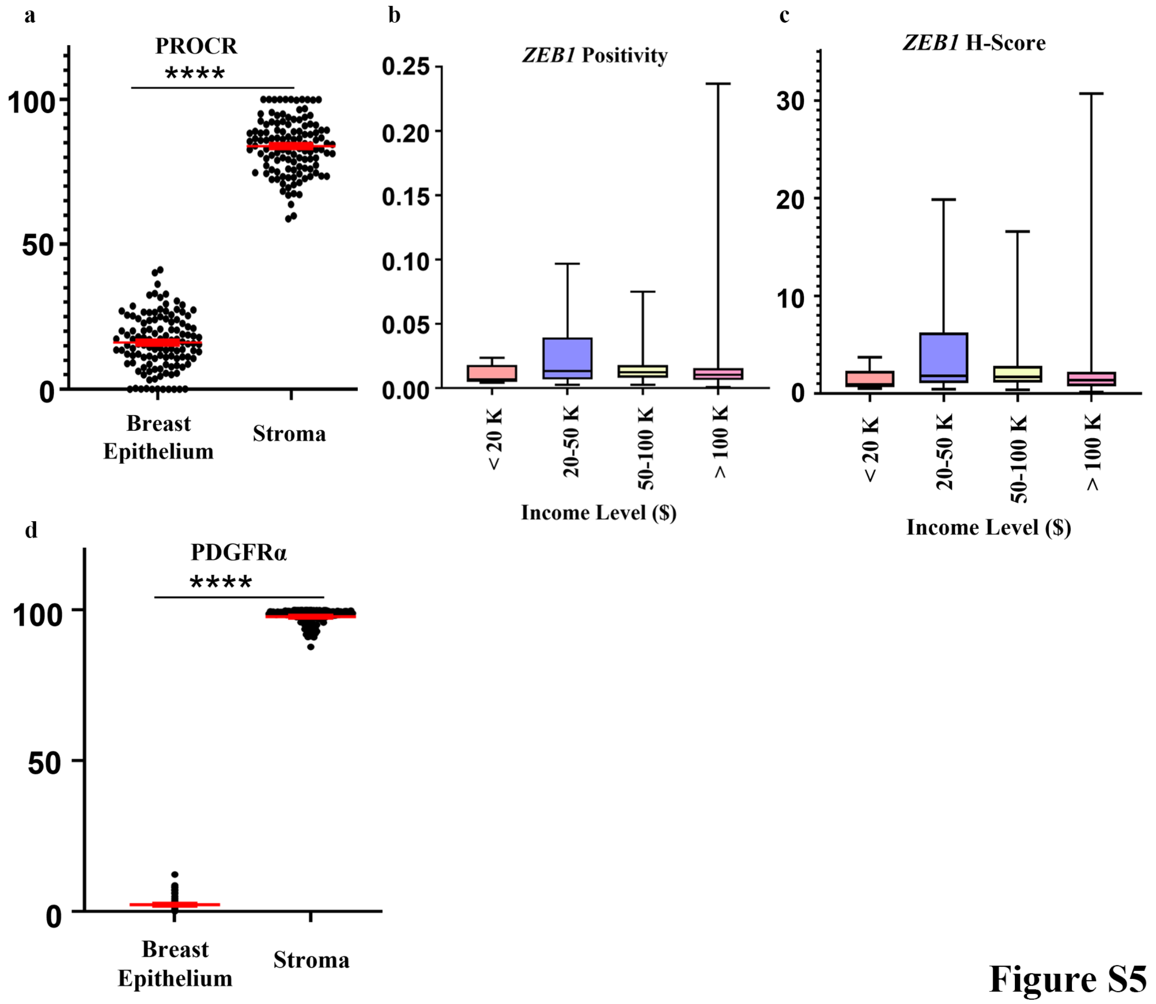

a
PROCR
****
100
50
0
Breast Epithelium
Stroma
b
ZEB1 Positivity
0.25
0.20
0.15
0.10
0.05
0.00
< 20 K
20-50 K
50-100 K
> 100 K
Income Level ($)
c
ZEB1 H-Score
30
20
10
0
< 20 K
20-50 K
50-100 K
> 100 K
Income Level ($)
d
PDGFRα
****
100
50
0
Breast Epithelium
Stroma

Figure S5

Figure S5: PROCR and PDGFRα expression patterns in breast epithelium and stroma of Normal-Healthy tissues. (a) PROCR expression was predominantly in stroma compared to breast epithelium (n=122). (b) Positivity of *ZEB1* in relation to income levels of donors, <20 K (n=12), 20-50 K (n=42), 50-100 K (n=65) and >100 K (n=73) in $. (c) H-score of *ZEB1* in relation to income levels of donors, <20 K (n=12), 20-50 K (n=42), 50-100 K (n=65) and >100 K (n=73) in $. Box plot representing whiskers with min-max data points and median value. (d) PDGFRα expression in stroma and breast epithelium (n=124). ****p<0.0001 analyzed using two tailed t-test. All the data points are shown as mean ± SEM. Source data are provided as a Source Data file.

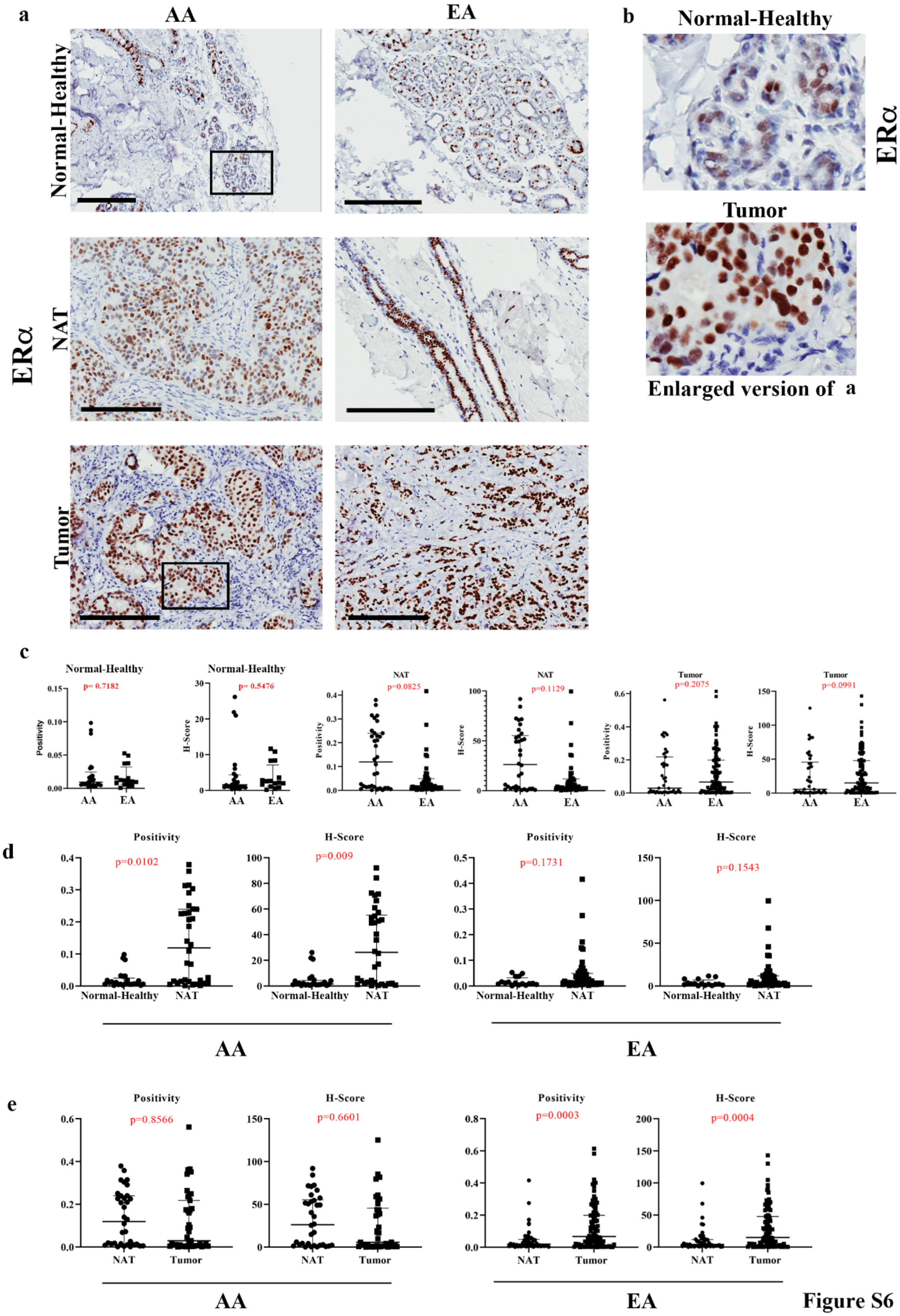
a
AA
EA
Normal-Healthy
NAT
Tumor
ERα
b
Normal-Healthy
ERα
Tumor
Enlarged version of a
c
Normal-Healthy
p= 0.7182
Positivity
AA
EA
Normal-Healthy
p= 0.5476
H-Score
NAT
p=0.0825
NAT
p=0.1129
Tumor
p=0.2075
Tumor
p=0.0991
d
Positivity
p=0.0102
H-Score
p=0.009
Positivity
p=0.1731
H-Score
p=0.1543
Normal-Healthy
NAT
e
Positivity
p=0.8566
H-Score
p=0.6601
Positivity
p=0.0003
H-Score
p=0.0004
NAT
Tumor

Figure S6

Figure S6: ERα expression pattern in Normal-Healthy, NATs, and breast tumors. (a) Representative IHC of ERα in Normal-Healthy, NATs, and tumors of women of African and European ancestry. (b) Enlarged view of ERα expression in Normal-Healthy and tumor. (c) Differences in ERα expression (positivity and H-score) between Normal-Healthy of breast tissues of women of African and European ancestry. Data analyzed using Wilcoxon test (Two-sided). (d) Differences in ERα expression (positivity and H-score) between Normal-Healthy and NATs in women of African and European ancestry. Data analyzed using two-tailed Wilcoxon test. (e) Differences in ERα expression (positivity and H-score) between NATs and tumors women of African and European ancestry. Data analyzed using two-tailed Wilcoxon test. (Normal-Healthy-AA (n=25), EA (n=16); NAT -AA (n=22), EA (n=34); Tumor -AA (n=29), EA (n= 59)). Dot blots contain data from duplicate cores of NATs and tumors wherever available. All the data points are shown as mean ± SD. Source data are provided as a Source Data file.

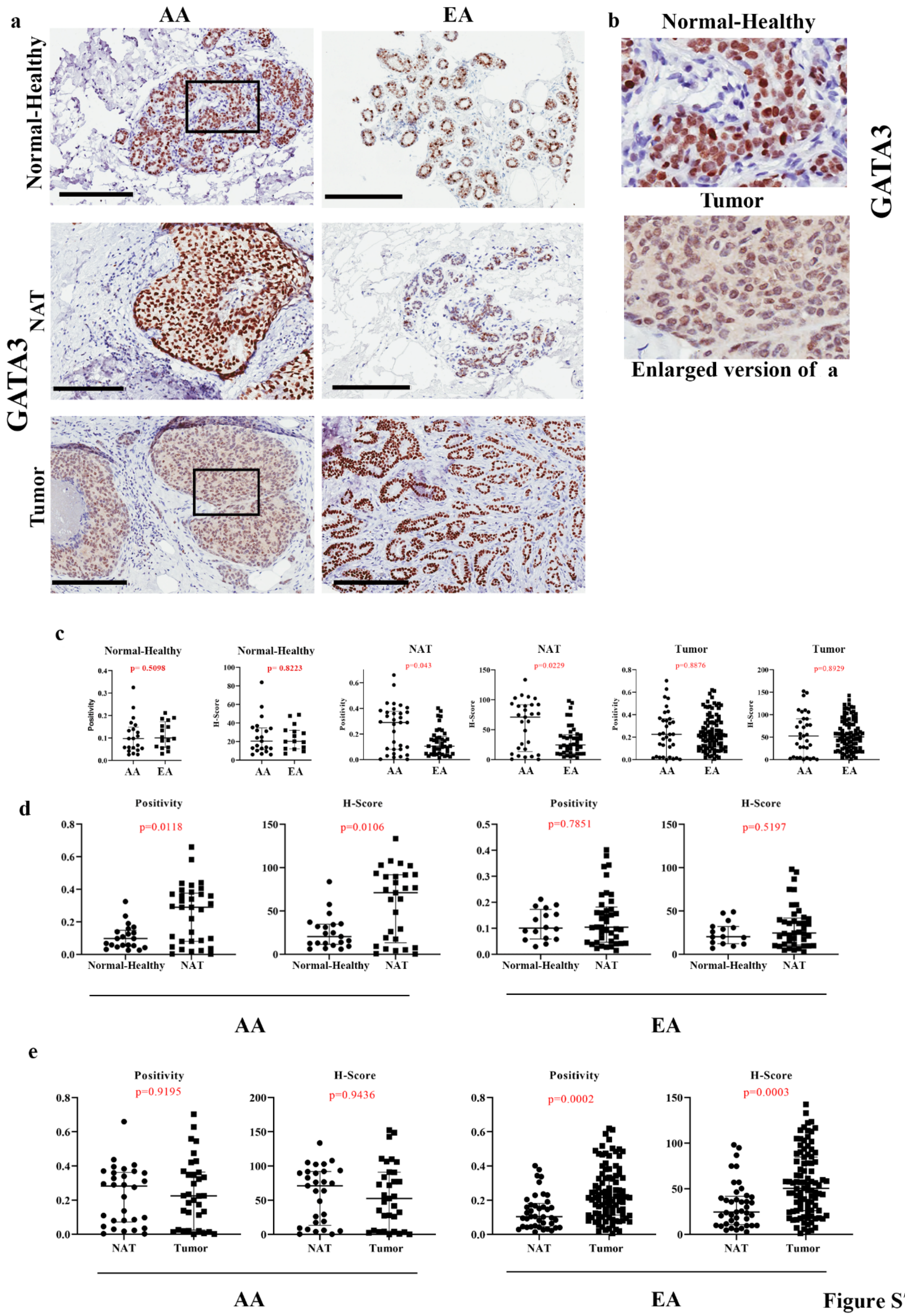
a
AA
EA
Normal-Healthy
NAT
Tumor
GATA3
b
Normal-Healthy
Tumor
GATA3
Enlarged version of a
c
Normal-Healthy
p= 0.5098
Normal-Healthy
p= 0.8223
NAT
p=0.043
NAT
p=0.0229
Tumor
p=0.8876
Tumor
p=0.8929
AA
EA
d
Positivity
p=0.0118
H-Score
p=0.0106
Positivity
p=0.7851
H-Score
p=0.5197
Normal-Healthy
NAT
AA
EA
e
Positivity
p=0.9195
H-Score
p=0.9436
Positivity
p=0.0002
H-Score
p=0.0003
NAT
Tumor
AA
EA

Figure S7

Figure S7: GATA3 expression pattern in Normal-Healthy, NATs, and breast tumors. (a) Representative IHC of GATA3 in Normal-Healthy, NATs, and tumors of women of African, and European ancestry. (b) Enlarged view of GATA3 expression in Normal-Healthy and tumor. (c) Differences in GATA3 expression (positivity and H-score) between Normal-Healthy of women of African and European ancestry. Data analyzed using Wilcoxon test (Two-sided). (d) Differences in GATA3 expression (positivity and H-score) between Normal-Healthy and NATs in women of African and European ancestry. Data analyzed using two-tailed Wilcoxon test. (e) Differences in GATA3 expression (positivity and H-score) between NATs and tumors in women of African and European ancestry. Data analyzed using two-tailed Wilcoxon test. (Normal-Healthy, AA (n=21), EA (n=16); NAT -AA (n=19), EA (n=29); Tumor -AA (n=23), EA (n= 58). Dot blots contain data from duplicate cores of NATs and tumors wherever available. All the data points are shown as mean ± SD. Source data are provided as a Source Data file.

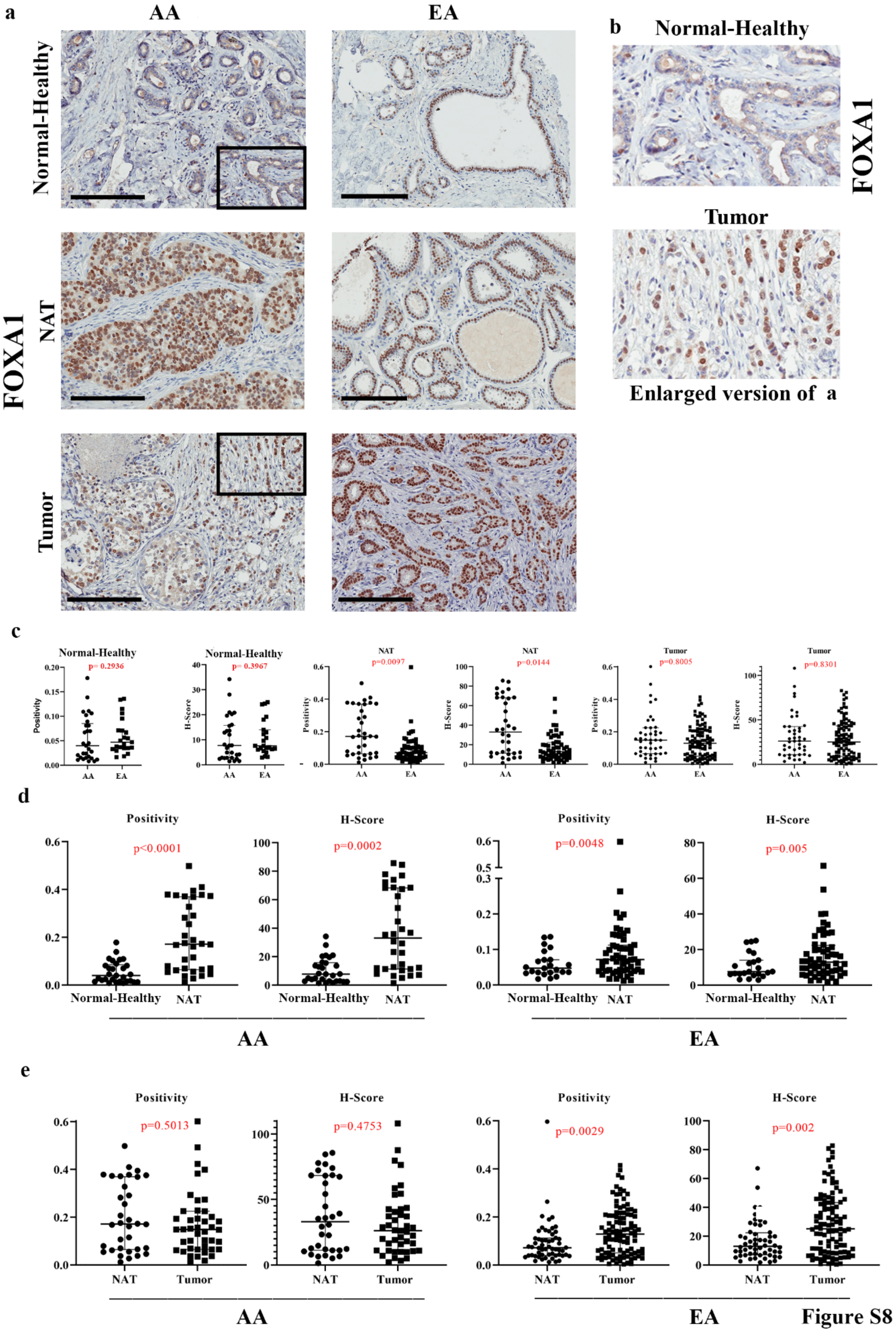
a
AA
EA
Normal-Healthy
FOXA1
NAT
Tumor
b
Normal-Healthy
FOXA1
Tumor
Enlarged version of a
c
Normal-Healthy
p= 0.2936
Positivity
AA
EA
Normal-Healthy
p= 0.3967
H-Score
NAT
p=0.0097
Positivity
NAT
p=0.0144
H-Score
Tumor
p=0.8005
Positivity
Tumor
p=0.8301
H-Score
d
Positivity
p<0.0001
H-Score
p=0.0002
Positivity
p=0.0048
H-Score
p=0.005
Normal-Healthy
NAT
AA
EA
e
Positivity
p=0.5013
H-Score
p=0.4753
Positivity
p=0.0029
H-Score
p=0.002
NAT
Tumor
AA
EA
Figure S8

Figure S8: FOXA1 expression pattern in Normal-Healthy, NATs, and breast tumors. (a) Representative IHC of FOXA1 in Normal-Healthy, NATs, and tumors of women of African, and European ancestry. (b) Enlarged view of FOXA1 expression in Normal-Healthy and tumor. (c) Differences in FOXA1 expression (positivity and H-score) between Normal-Healthy of women of African, and European ancestry. Data analyzed using Wilcoxon test (Two-sided). (d) Differences in FOXA1 expression (positivity*p=0.000079348 and H-score) between Normal-Healthy and NATs in women of African and European ancestry. Data analyzed using two-tailed Wilcoxon test. (e) Differences in FOXA1 expression (positivity and H-score) between NATs and tumors in women of African and European ancestry. Data analyzed using two-tailed Wilcoxon test. (Normal-Healthy, AA (n=29), EA (n=23); NAT -AA (n=22), EA (n=38); Tumor -AA (n=26), EA (n= 59). Dot blots contain data from duplicate cores of NATs and tumors wherever available. All the data points are shown as mean ± SD. Source data are provided as a Source Data file.

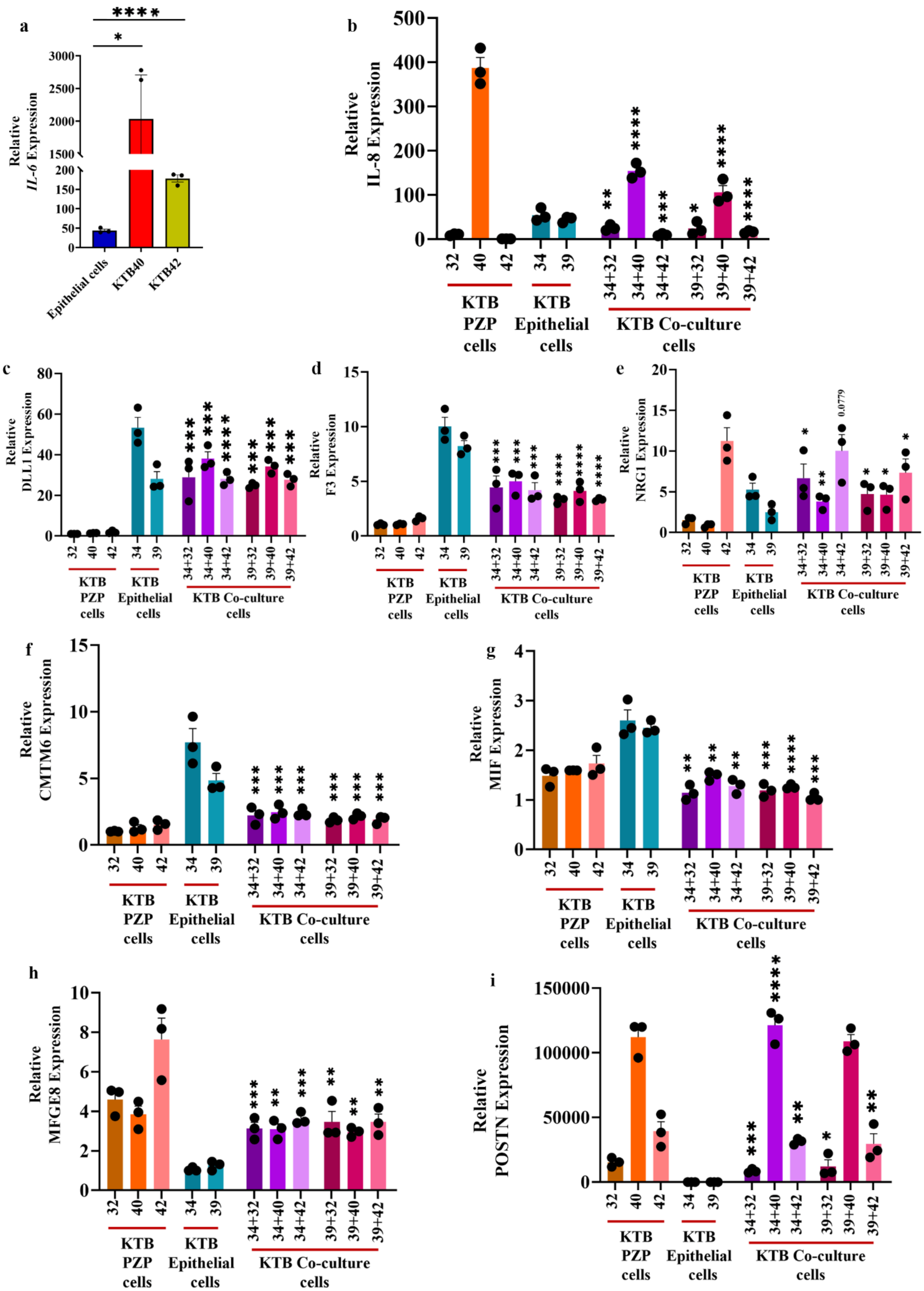
a
Relative
IL-6 Expression
****
*
Epithelial cells
KTB40
KTB42
b
Relative
IL-8 Expression
KTB
PZP
cells
KTB
Epithelial
cells
KTB Co-culture
cells
c
Relative
DLL1 Expression
d
Relative
F3 Expression
e
Relative
NRG1 Expression
0.0779
f
Relative
CMTM6 Expression
g
Relative
MIF Expression
h
Relative
MFGE8 Expression
i
Relative
POSTN Expression
32
40
42
34
39
34+32
34+40
34+42
39+32
39+40
39+42

Figure S9

Figure S9: The effects of co-culture of PZP and luminal progenitor cell lines on expression of specific genes. (a) Relative mRNA level of IL-6 in KTB40, KTB42 and KTB-epithelial cell lines (n=3). Data analyzed using two tailed t-test. (KTB40*p=0.0417; KTB42*p<0.0001) Expression of (b) IL-8 (n=3) (KTB34+32*p=0.0033, KTB34+40*p<0.0001; KTB34+42*p=0.0005; KTB39+32*p=0.0111; KTB39+40*p<0.0001; KTB39+42*p<0.0001), (c) DLL1 (n=3) (KTB34+32*p=0.0006, KTB34+40*p=0.0001; KTB34+42*p<0.0001; KTB39+32*p=0.0002; KTB39+40*p=0.0001; KTB39+42*p=0.0003),

(d) F3 (n=3) (KTB34+32*p=0.0005, KTB34+40*p=0.0002; KTB34+42*p=0.0002; KTB39+32*p<0.0001; KTB39+40*p<0.0001; KTB39+42*p<0.0001),

(e) NRG1 (n=3) (KTB34+32*p=0.04, KTB34+40*p=0.0038; KTB39+32*p=0.0440; KTB39+40*p=0.0158; KTB39+42*p=0.0137),

(f) CMTM6 (n=3) (KTB34+32*p=0.0006, KTB34+40*p=0.0008; KTB34+42*p=0.0008; KTB39+32*p=0.0003; KTB39+40*p=0.0008; KTB39+42*p=0.0009),

(g) MIF (n=3) (KTB34+32*p=0.0011, KTB34+40*p=0.0015; KTB34+42*p=0.0036; KTB39+32*p=0.0001; KTB39+40*p<0.0001; KTB39+42*p=0.0003),

(h) MFGE8 (n=3) (KTB34+32*p=0.0005, KTB34+40*p=0.0012; KTB34+42*p=0.0009; KTB39+32*p=0.003; KTB39+40*p=0012; KTB39+42*p=0.0014), and

(i) POSTN (n=3) (KTB34+32*p=0.0004, KTB34+40*p<0.0001; KTB34+42*p=0.0014; KTB39+32*p=0.0287; KTB39+40*p<0001; KTB39+42*p=0.0097) in PZP cell lines (KTB32, KTB40, and KTB42), luminal progenitor (epithelial cells; KTB34 and KTB39), and co-culture of PZP and luminal progenitor cell lines. Statistical significance (*p* values) was determined by comparing KTB32/40/42 (PZP cells), KTB34/KTB39 (epithelial cells), and respective co-cultured

PZP + epithelial cells as indicated in the figure. All the data points are shown as mean ± SEM. Source data are provided as a Source Data file.

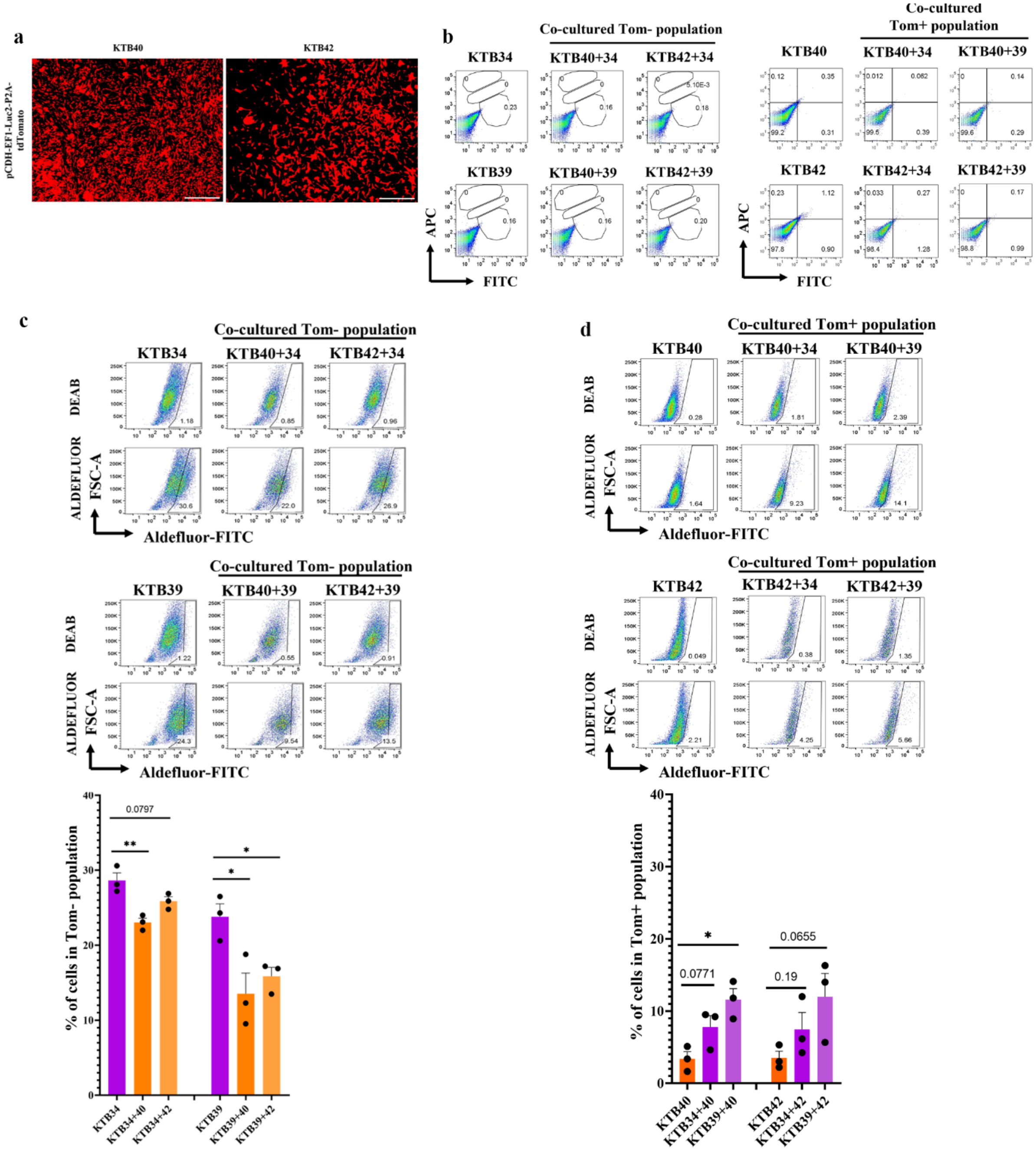
a
KTB40
KTB42
pCDH-EF1-Luc2-P2A-tdTomato
b
Co-cultured Tom- population
KTB34
KTB40+34
KTB42+34
KTB39
KTB40+39
KTB42+39
APC
FITC
Co-cultured Tom+ population
KTB40
KTB40+34
KTB40+39
KTB42
KTB42+34
KTB42+39
c
Co-cultured Tom- population
KTB34
KTB40+34
KTB42+34
DEAB
ALDEFLUOR
FSC-A
Aldefluor-FITC
KTB39
KTB40+39
KTB42+39
% of cells in Tom- population
0.0797
KTB34
KTB34+40
KTB34+42
KTB39
KTB39+40
KTB39+42
d
Co-cultured Tom+ population
KTB40
KTB40+34
KTB40+39
KTB42
KTB42+34
KTB42+39
% of cells in Tom+ population
0.0771
0.0655
0.19
KTB40
KTB34+40
KTB39+40
KTB42
KTB34+42
KTB39+42

Figure S10

Figure S10: The effects of PZP cells on trans-differentiation of epithelial cells (a) PZP KTB40 and KTB42 cells were labelled with tomato-red using pCDH-EF1-Luc2-P2A-tdTomato lentivirus and sorted tomato-red positive cells by flow cytometry to generate stable cell lines. (n=1) (b) APC and FITC isotypes control staining patterns of cell lines is shown. These staining patterns were used to draw quadrants. (c) ALDEFLUOR staining patterns of KTB34, KTB39, and co-cultured KTB40/KTB42 and KTB34/KTB39 cell lines (n=3). Quantification of $ALDH^+$ cells in Tom^- cell epithelial cell population (n=3). (KTB40+34*p=0.0087; KTB40+39*p=0.034, KTB42+39*p= 0.0192). (d) ALDEFLUOR staining patterns of KTB40, KTB42, and co-cultured KTB40/KTB42 and KTB34/KTB39 cell lines. Only Tom^+ PZP cells were analyzed (n=3). Quantification of $ALDH^+$ cells among Tom^+ population (n=3) (KTB42+34*p=0.0102). *p<0.05, **p<0.01). The data analyzed using two tailed t-test. All the data points are shown as mean ± SEM. Source data are provided as a Source Data file.

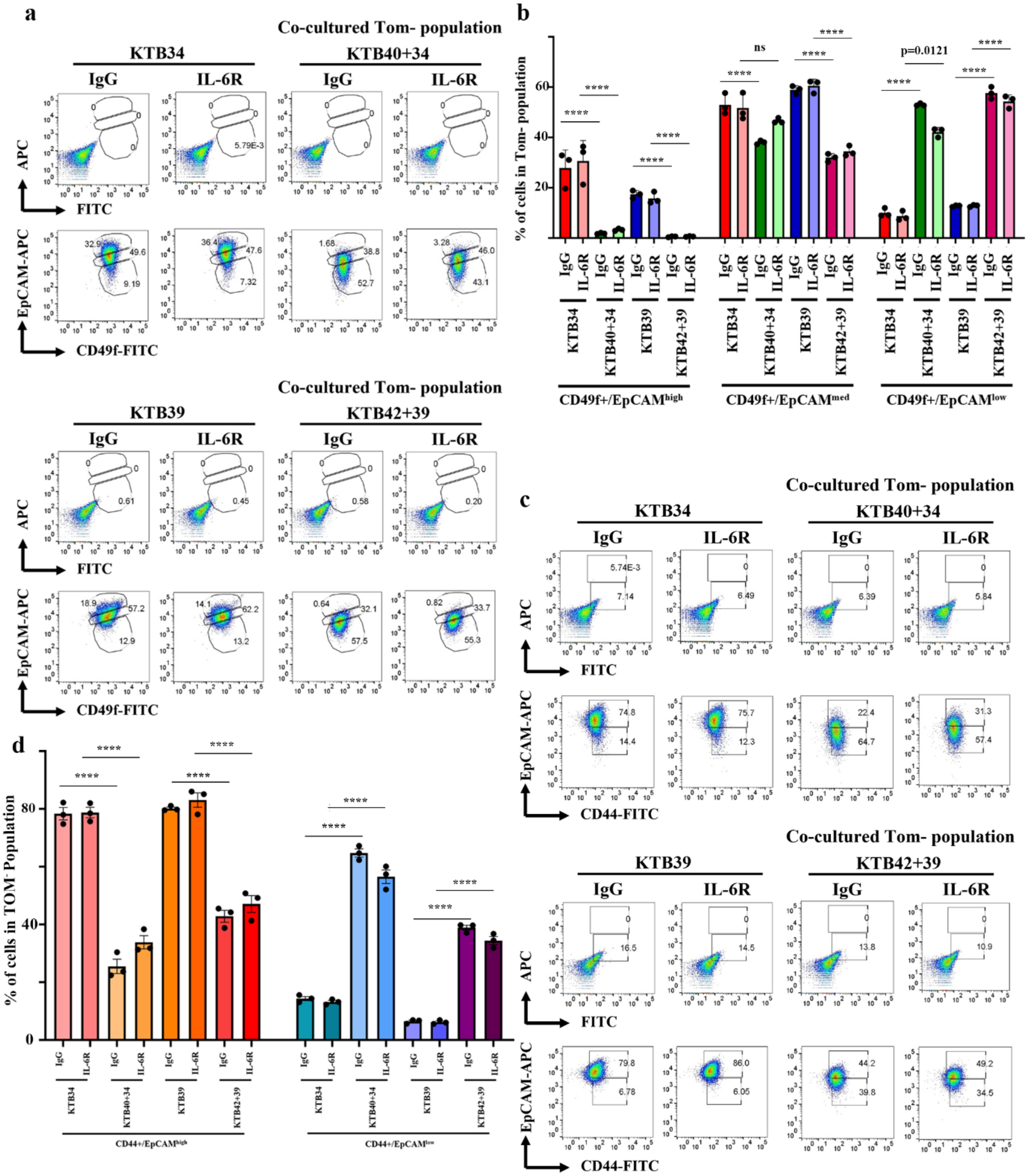
a
Co-cultured Tom- population
KTB34
KTB40+34
IgG
IL-6R
APC
FITC
EpCAM-APC
CD49f-FITC
KTB39
KTB42+39
b
% of cells in Tom- population
CD49f+/EpCAMhigh
CD49f+/EpCAMmed
CD49f+/EpCAMlow
ns
p=0.0121
c
CD44-FITC
d
% of cells in TOM- Population
CD44+/EpCAMhigh
CD44+/EpCAMlow

Figure S11

Figure S11: The effects of IL-6 neutralization on PZP cell-induced trans-differentiation of epithelial cells. (a) Treatment of co-cultured KTB34 and KTB40 cells with neutralizing IL-6R antibody showed a decrease in $CD49f^{+}/EpCAM^{-/low}$ population compared to co-cultured IgG control (n=3). KTB39 and KTB42 co-cultured cells followed by neutralizing IL-6R antibody treatment showed a modest decrease in $CD49f^{+}/EpCAM^{-/low}$ population (n=3). (b) Quantification of $CD49f^{+}/EpCAM^{high}$, $CD49f^{+}/EpCAM^{med}$, and $CD49f^{+}/EpCAM^{low}$ populations in Tom^{-} cell population (n=3). The effect of IL-6R antibody on trans-differentiation was significant (p=0.0121, two-way ANOVA). (c) Analysis of $CD44^{+}/EpCAM^{high}$ and $CD44^{+}/EpCAM^{low}$ populations in KTB34, co-cultured KTB34 and KTB40, KTB39, and co-cultured KTB39 and KTB42 cells with IgG control and neutralizing IL-6R antibody treatment (n=3). (d) Quantification of $CD44^{+}/EpCAM^{high}$ and $CD44^{+}/EpCAM^{low}$ subpopulations in Tom^{-} KTB34 and KTB39 cells (n=3). The data analyzed using two-way ANOVA, ****$p<0.0001$ for all. While the effects of PZP cells in altering CD44/EpCAM staining patterns of epithelial cells were significant (**$p<0.0001$ by two-way ANOVA), the effects of IL-6R in reversing PZP-induced changes in epithelial cell CD44/EpCAM staining patterns were not significant. All the data points are shown as mean ± SEM. Source data are provided as a Source Data file.

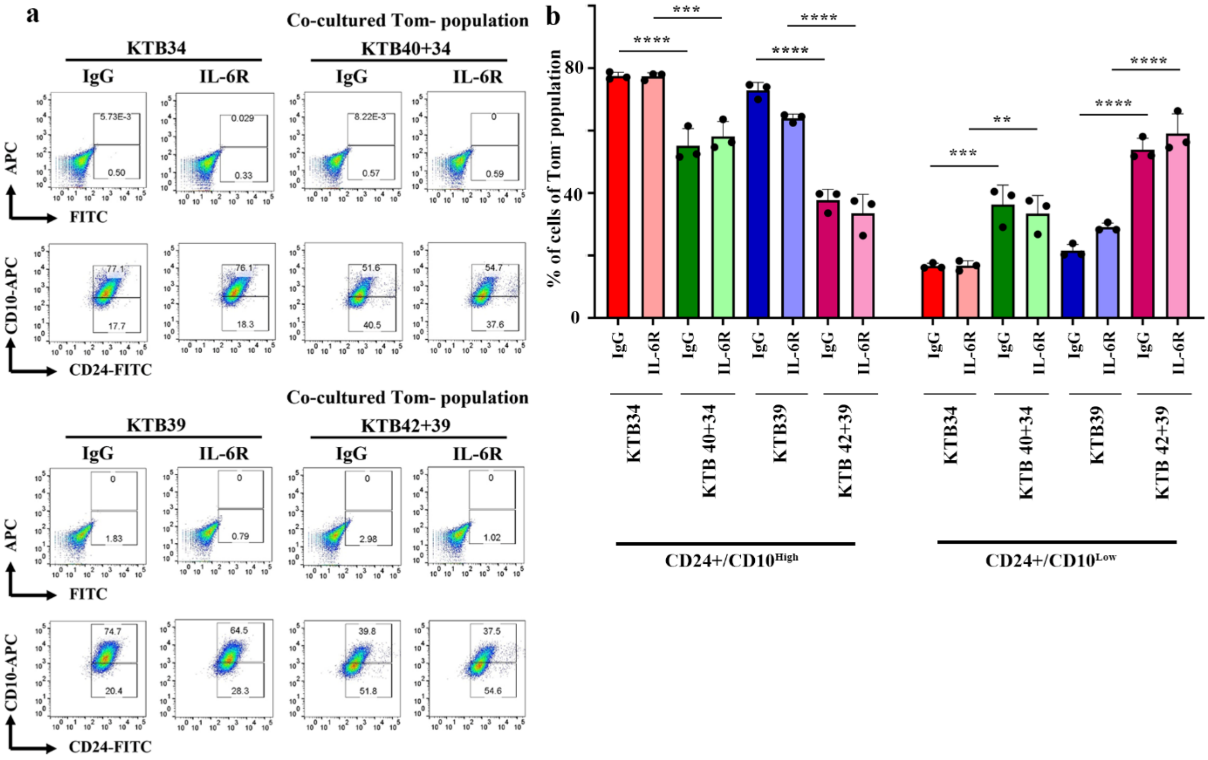
a
Co-cultured Tom- population
KTB34
KTB40+34
IgG
IL-6R
IgG
IL-6R
APC
FITC
5.73E-3
0.50
0.029
0.33
8.22E-3
0.57
0
0.59
CD10-APC
CD24-FITC
77.1
17.7
76.1
18.3
51.6
40.5
54.7
37.6
Co-cultured Tom- population
KTB39
KTB42+39
IgG
IL-6R
IgG
IL-6R
APC
FITC
0
1.83
0
0.79
0
2.98
0
1.02
CD10-APC
CD24-FITC
74.7
20.4
64.5
28.3
39.8
51.8
37.5
54.6
b
% of cells of Tom⁻ population
80
40
0
****
***
****
****
***
**
****
****
IgG
IL-6R
KTB34
KTB 40+34
KTB39
KTB 42+39
CD24+/CD10High
CD24+/CD10Low

Figure S12

Figure S12: The effects of IL-6 neutralization on CD24 and CD10 staining patterns of epithelial cells. (a) Analysis of $CD24^+/CD10^{high}$ and $CD24^+/CD10^{low}$ populations in KTB34, co-cultured KTB34 and KTB40, KTB39, and co-cultured KTB39 and KTB42 cells with IgG control or neutralizing IL-6R antibody treatment (n=3). (b) Quantification of $CD24^+/CD10^{high}$ (KTB34*p<0.0001; KTB40+34*p=0.0002; KTB39*p<0.0001; KTB42+39*p<0.0001) and $CD24^+/CD10^{low}$ (KTB34*p=0.0002; KTB40+34*p=0.0018; KTB39*p<0.0001; KTB42+39*p<0.0001) populations in Tom^- cell population (n=3). While the co-culture-induced changes in CD24 and CD10 staining patterns were significant (**p<0.0018, ***p<0.0002, ****p<0.0001), the effects of IL-6R antibody in reversing these changes were not significant. The data analyzed using two-way ANOVA. All the data points are shown as mean ± SEM. Source data are provided as a Source Data file.

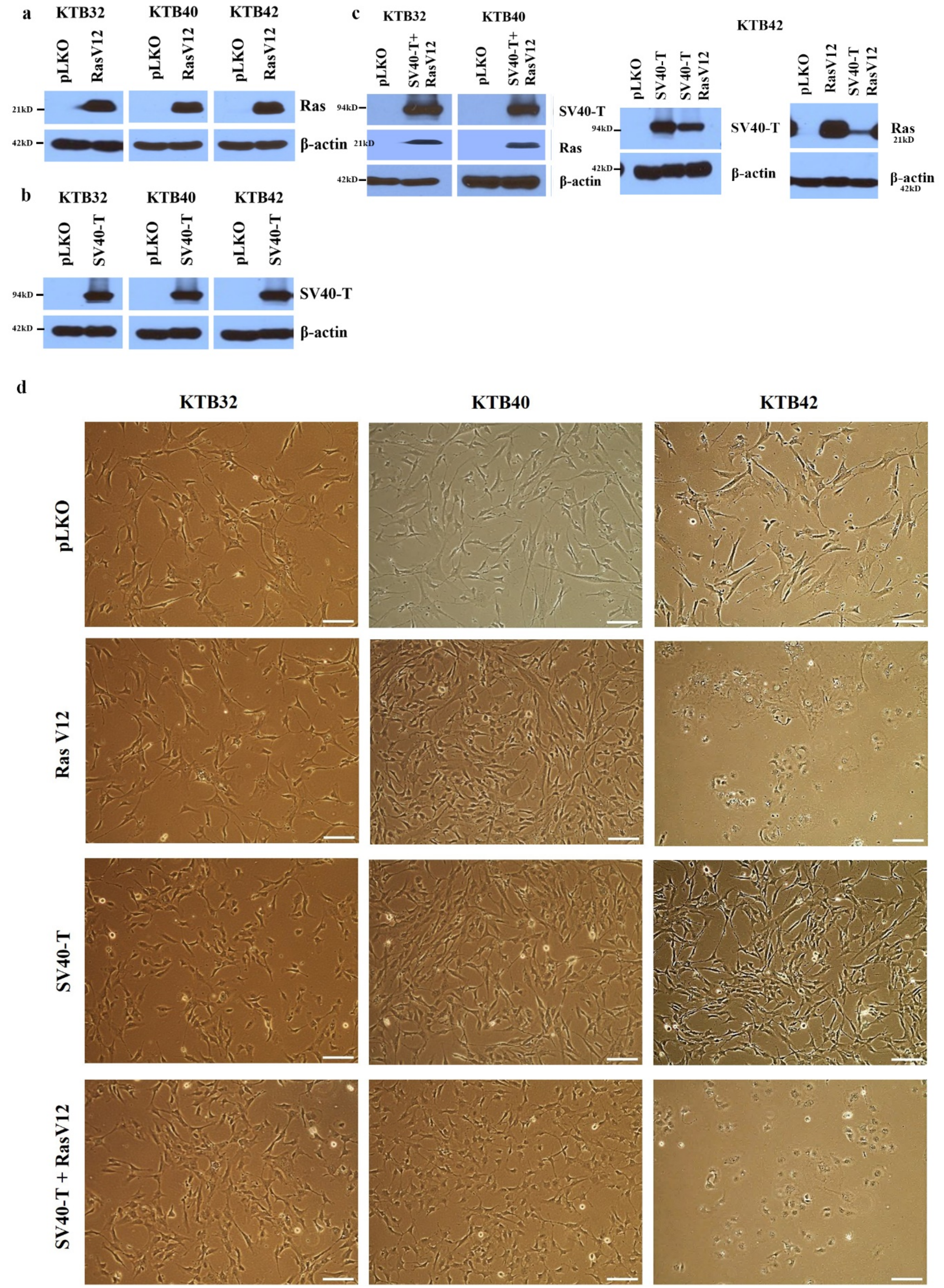
a
KTB32
KTB40
KTB42
pLKO
RasV12
Ras
21kD
42kD
β-actin
b
SV40-T
94kD
c
SV40-T+ RasV12
SV40-T RasV12
d
Ras V12
SV40-T + RasV12

Figure S13

Figure S13: Generation of $HRas^{G12V}$, SV40-T/t antigen and $HRas^{G12V}$+ SV40-T/t antigen transformed PZP cell lines. (a) Western blotting was used to detect overexpression of mutant Ras in KTB32, KTB40, and KTB42 cell lines. (n=1) (b) Western blotting was used to detect overexpression of SV40-T/t antigens in KTB32, KTB40, and KTB42 cell lines. (n=1) (c) Western blotting was used to detect overexpression of mutant Ras and SV40-T/t antigens in double transformed KTB32, KTB40 and KTB42 cell lines. Empty vector pLKO containing cell line was used as a control cell line. β–actin was used as an internal control. (n=1) (d) Phase contrast images showing morphology of KTB32, KTB40, and KTB42 cell lines transformed with $HRas^{G12V}$, SV40-T/t antigens, and $HRas^{G12V}$+ SV40-T/t antigen. (n=1).

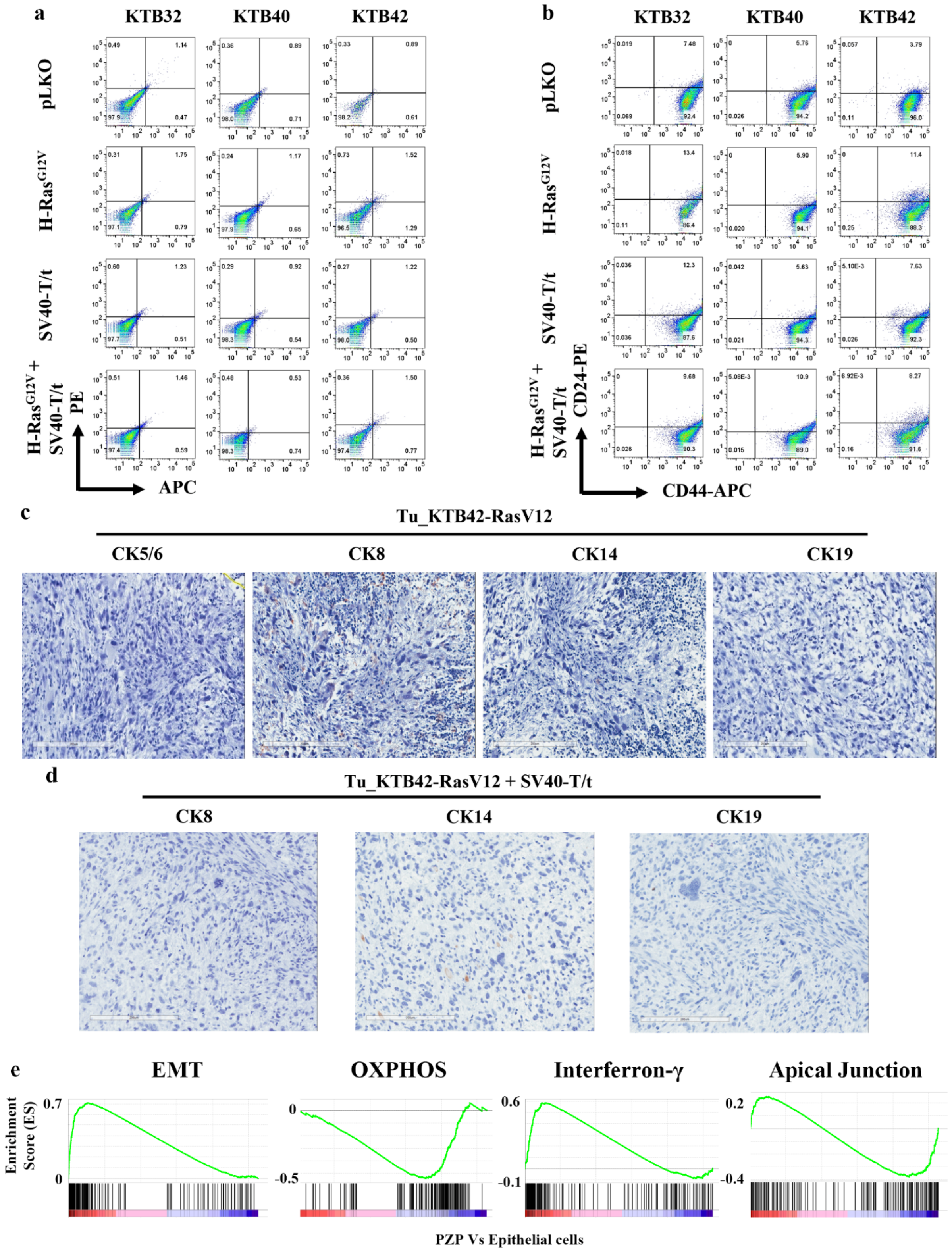
a
KTB32
KTB40
KTB42
pLKO
H-Ras^G12V
SV40-T/t
H-Ras^G12V + SV40-T/t
PE
APC
b
KTB32
KTB40
KTB42
pLKO
H-Ras^G12V
SV40-T/t
H-Ras^G12V + SV40-T/t
CD24-PE
CD44-APC
c
Tu_KTB42-RasV12
CK5/6
CK8
CK14
CK19
d
Tu_KTB42-RasV12 + SV40-T/t
CK8
CK14
CK19
e
EMT
OXPHOS
Interferron-γ
Apical Junction
Enrichment Score (ES)
PZP Vs Epithelial cells

Figure S14

Figure S14: Characterization of transformed cell lines and tumors. (a) APC and PE isotypes controls staining of cell lines (n=3). These staining patterns were used to draw quadrants. (b) CD44 and CD24 staining patterns of immortalized and transformed PZP (KTB32, KTB40 and KTB42) cell lines (n=3). (c) IHC analyses of cytokeratins CK5/6, CK8, CK14, and CK19 in tumors developed from KTB42-HRas^{G12V} transformed cells. Basal markers: CK5/6 and CK14, Luminal markers: CK8 and CK19 (n=5). (d) IHC analyses of cytokeratins CK8, CK14, and CK19 in tumors developed from the KTB42-HRas^{G12V}+SV40-T/t antigen transformed cells (n=5). (e) GSEA analysis of genes differentially expressed in PZP cells compared to epithelial cells reveals enrichment of signaling networks in PZP cells similar to that of metaplastic carcinomas of the breast.

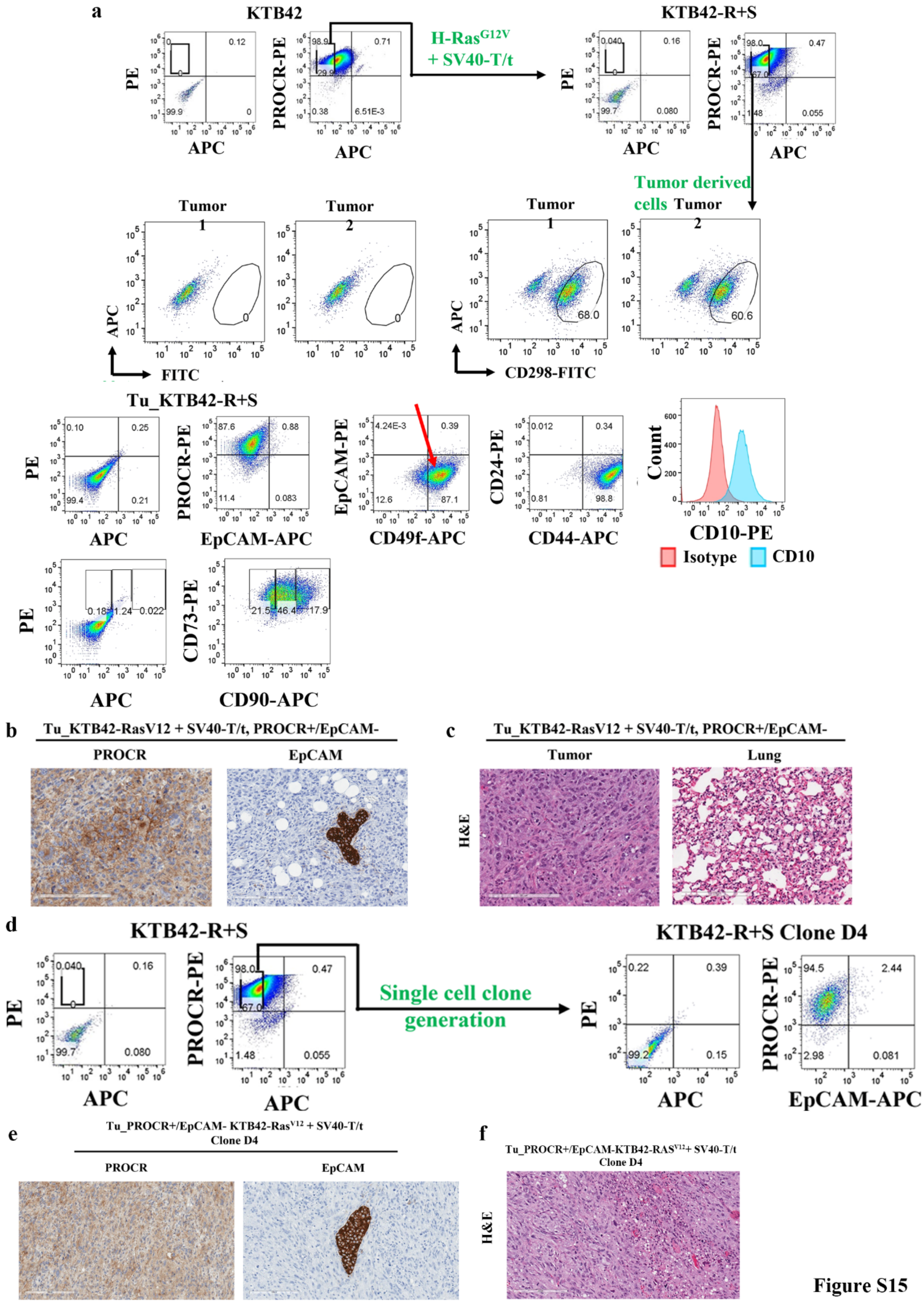
a
KTB42
H-Ras^{G12V} + SV40-T/t
KTB42-R+S
Tumor derived cells
Tumor 1
Tumor 2
APC
FITC
CD298-FITC
Tu_KTB42-R+S
PE
PROCR-PE
EpCAM-APC
EpCAM-PE
CD49f-APC
CD24-PE
CD44-APC
Count
CD10-PE
Isotype
CD10
CD73-PE
CD90-APC
b
Tu_KTB42-RasV12 + SV40-T/t, PROCR+/EpCAM-
PROCR
EpCAM
c
Tu_KTB42-RasV12 + SV40-T/t, PROCR+/EpCAM-
Tumor
Lung
H&E
d
KTB42-R+S
Single cell clone generation
KTB42-R+S Clone D4
e
Tu_PROCR+/EpCAM- KTB42-Ras^{V12} + SV40-T/t Clone D4
PROCR
EpCAM
f
Tu_PROCR+/EpCAM-KTB42-RAS^{V12}+ SV40-T/t Clone D4
H&E

Figure S15

Figure S15: Characterization of tumors developed from PROCR^{+}/EpCAM^{-} KTB42 cells transformed with HRas^{G12V} and SV40-T/t antigens. (a) PROCR^{+}/EpCAM^{-} cells were sorted from KTB42-HRas^{G12V}+SV40-T/t transformed cells and injected into NSG mice that formed the tumors. Human cells were isolated from tumors by flow cytometry using CD298 marker to establish tumor-derived cell lines. Tumor-derived cell lines were characterized using cell surface markers (PROCR, EpCAM, CD49f, CD44, CD24, CD10, CD73, and CD90) by flow cytometry (n=3). (b) IHC analyses of PROCR and EpCAM in tumors developed from PROCR^{+}/EpCAM^{-} KTB42-HRas^{G12V}+SV40-T/t transformed cells (n=6). (c) Tumor developed from PROCR^{+}/EpCAM^{-} KTB42-HRas^{G12V}+SV40-T/t transformed cells did not show lung metastasis (n=6). H&E staining shows tumor phenotype. (d) Single cell was sorted from PROCR^{+}/EpCAM^{-} population of KTB42-HRas^{G12V}+SV40-T/t transformed cells and plated into 96 well plate. Clone D4 from single cell was established and characterized for PROCR and EpCAM markers. (e) IHC analyses of tumors (n=5) derived from clone D4 cells for PROCR and EpCAM. (f) H&E staining shows tumor phenotype (n=5).

a Gating strategy applied for Fig. 1b.S1d,3a,3d,3h,3j,S2

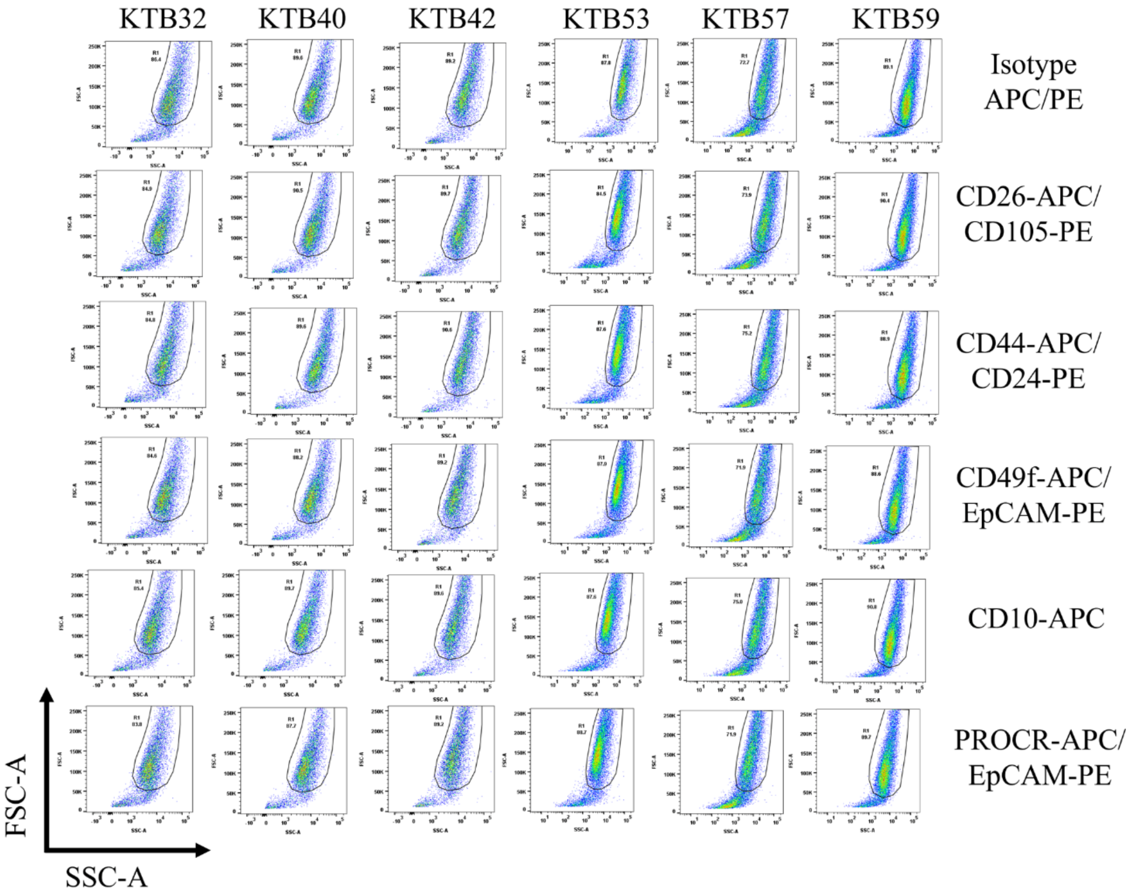

b Gating Strategy applied for Fig. 1e, S1f

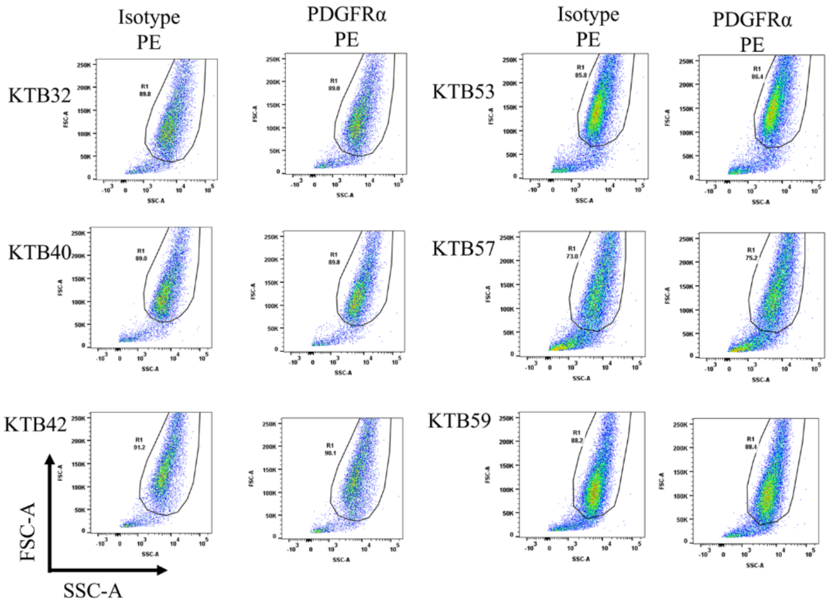

c **Gating Strategy for Fig. S1, S2**

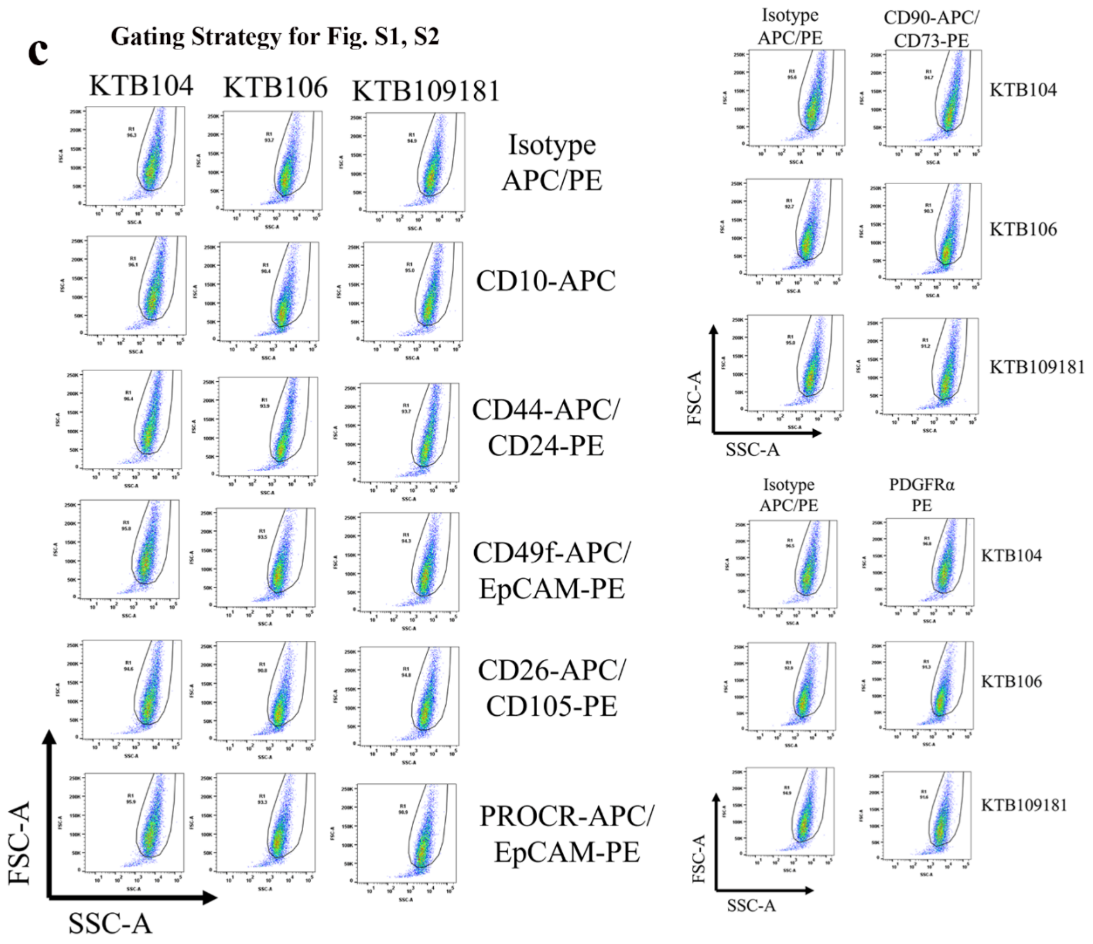

d **Gating Strategy applied for Fig. 8, S10**

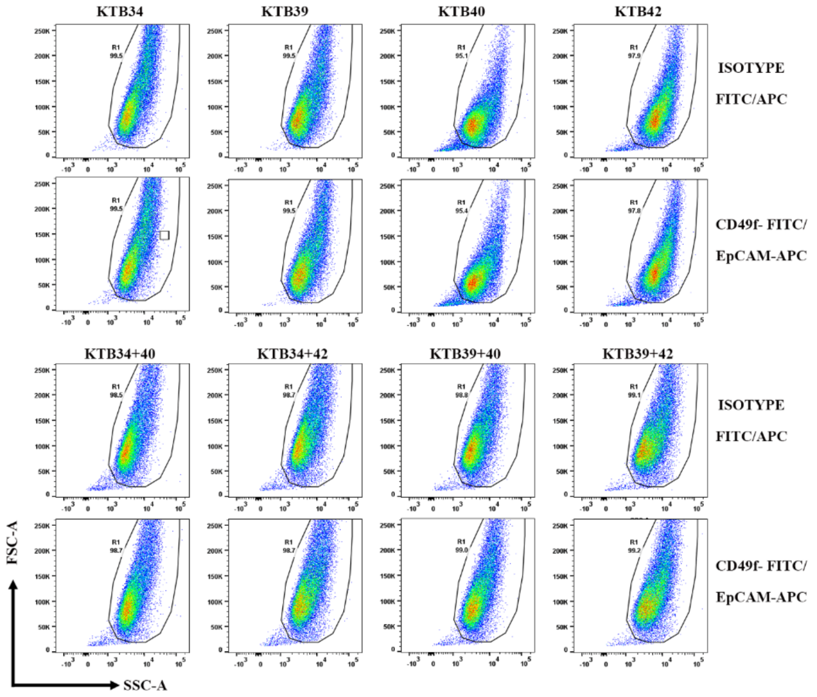

e

Gating Strategy applied for Fig. 10a-c,S14a-b

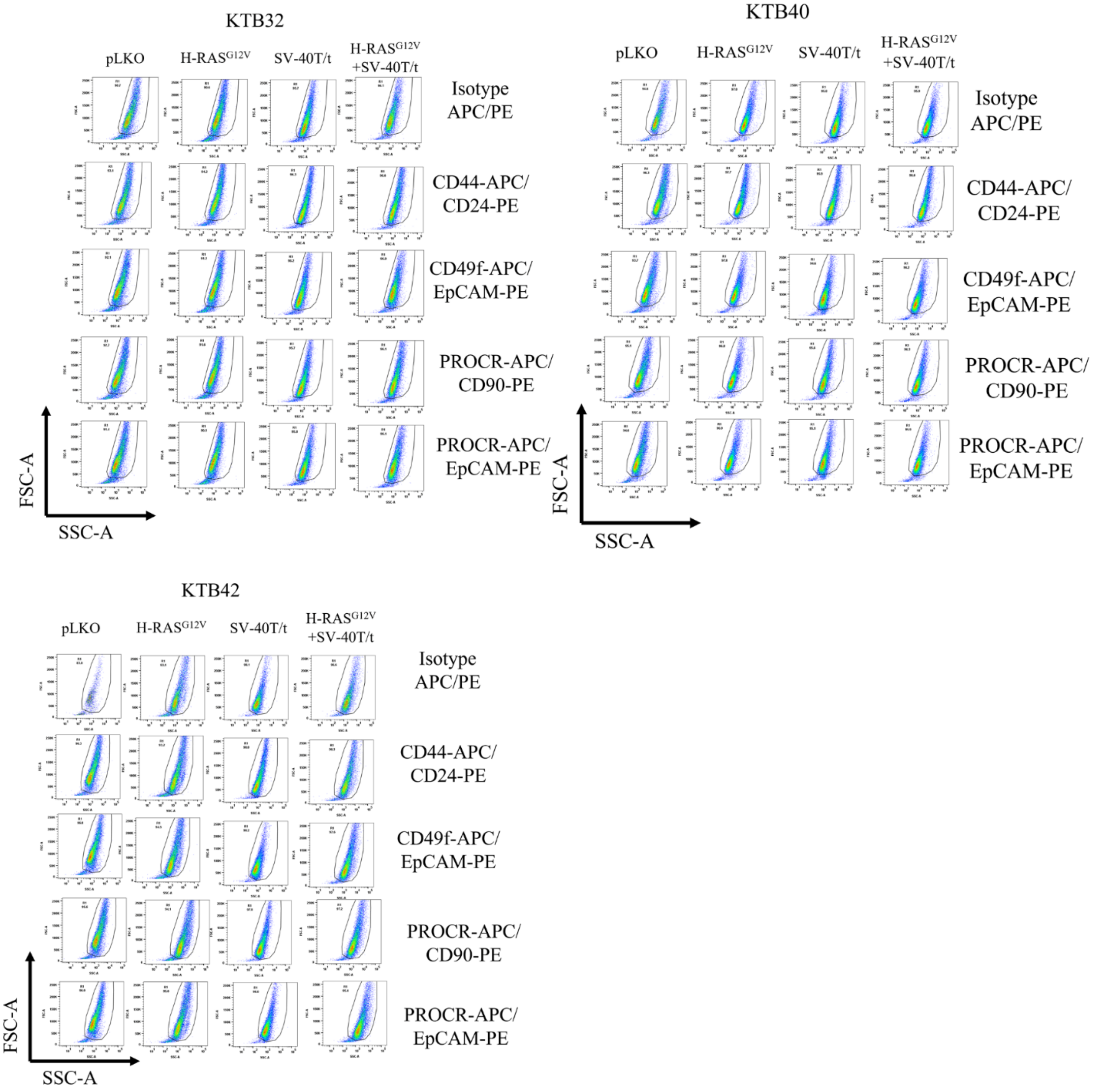

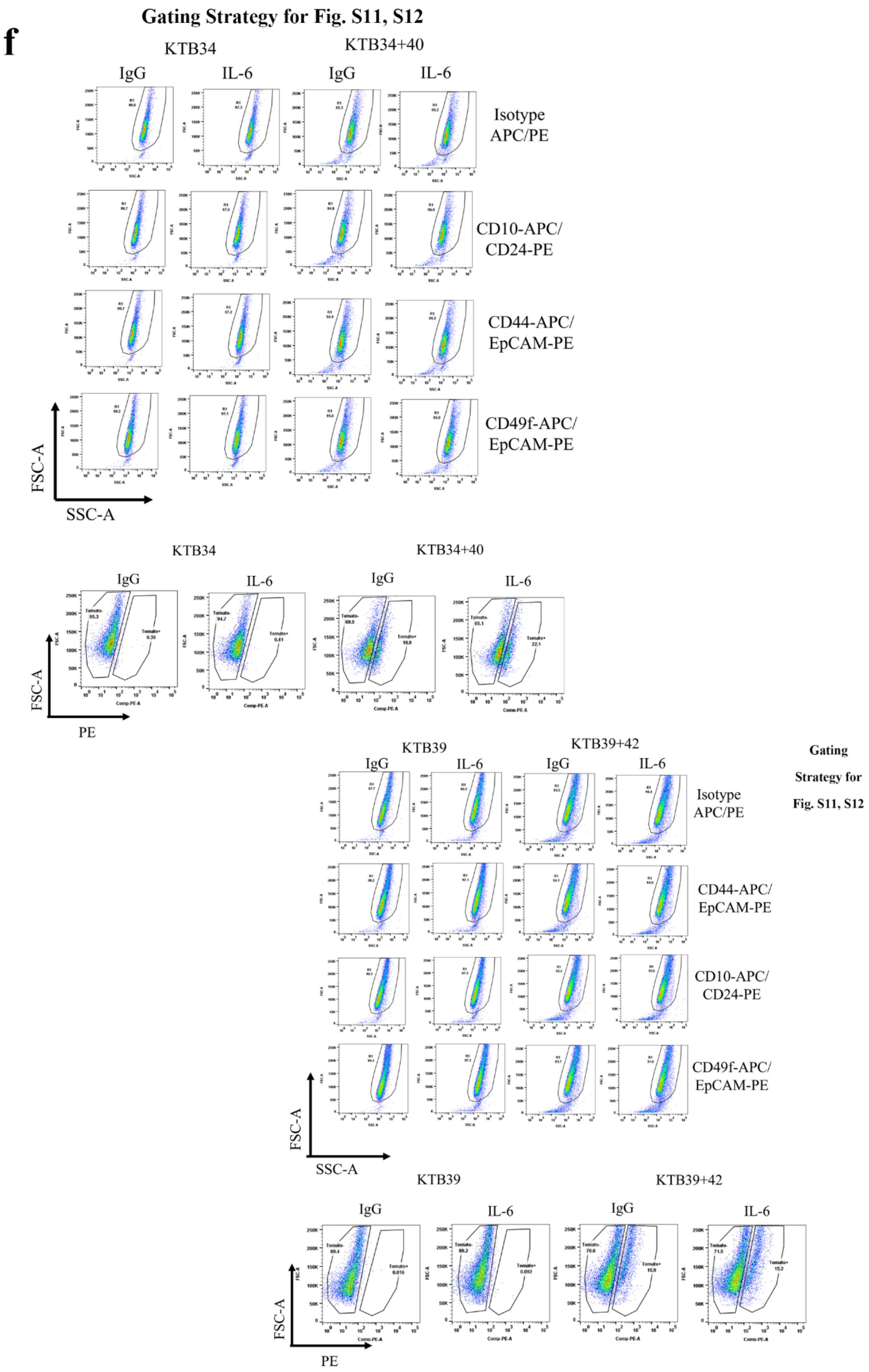
f
Gating Strategy for Fig. S11, S12
KTB34
KTB34+40
IgG
IL-6
IgG
IL-6
Isotype APC/PE
CD10-APC/ CD24-PE
CD44-APC/ EpCAM-PE
CD49f-APC/ EpCAM-PE
FSC-A
SSC-A
KTB34
KTB34+40
IgG
IL-6
IgG
IL-6
FSC-A
PE
KTB39
KTB39+42
IgG
IL-6
IgG
IL-6
Isotype APC/PE
CD44-APC/ EpCAM-PE
CD10-APC/ CD24-PE
CD49f-APC/ EpCAM-PE
Gating Strategy for Fig. S11, S12
FSC-A
SSC-A
KTB39
KTB39+42
IgG
IL-6
IgG
IL-6
FSC-A
PE

Figure S16: Gating Strategy for flow cytometry analysis (a-f) Forward and side scatter plots of figures represented in main text.

Table S1: Histopathological features of tumors used in the TMA.

	AA	EA
ER+/PR+	20	68
ER+/PR-	3	12
ER-/PR+	0	3
ER+/PR+/HER2+	3	16
ER+/HER2+	3	6
HER2+	6	7
TNBC	8	11
NA	6	13
$Node^{+}$	16	20
Grade 1	1	10
Grade 2	11	69
Grade 3	23	36
Stage 1	0	5
Stage 2	9	17
Stage 3	1	6
Stage 4	1	1
No data available	5	1
Total Samples	49	136

Table S2. Compare H-score and Positivity between genetic ancestries within Normal-Heathy tissues for PROCR, ZEB1, and PDGFRα

Marker		Genetic Ancestry			
	Variable Label	Column_Overall	African American	European	P-value
**PROCR**	Positivity	0.17 (0.04, 0.55)	0.30 (0.06, 0.55) (N=31)	0.15 (0.04, 0.41) (N=129)	2.2051E-8
	H-Score	27.97 (5.45, 127.65)	56.41 (10.46, 127.65)	24.37 (6.68, 83.50)	6.0538E-9
**ZEB1**	Positivity	0.01 (0.00, 0.24)	0.01 (0.00, 0.10) (N=33)	0.01 (0.00, 0.12) (N=144)	0.0739
	H-Score	1.62 (0.15, 30.72)	2.21 (0.24, 16.47)	1.46 (0.15, 19.76)	0.0249
**PDGFRα**	Positivity	0.10 (0.01, 0.75)	0.28 (0.03, 0.75) (N=35)	0.09 (0.01, 0.73) (N=154)	0.000000251
	H-Score	16.30 (1.43, 110.23)	36.96 (6.25, 110.23)	14.27 (1.43, 88.61)	0.000014339

Note: Values expressed as median (min, max)

Note: P-value comparisons across race categories are based on Two-sided Wilcoxon test.

Table S3. Cytokine/chemokine array identified factors secreted by luminal and PZP cells either alone or together.

Shared	Angiogenin, Angiopoietin 2, BDNF, DKK1, Emmprin, IFNγ, IGFBP2, IGFBP3, IL-8, Kallikrein 3, MIF, Osteopontin, Pentraxin 3, Serpin E1, ST2 and VEGF
Luminal and in co-culture	GDF15, GROα, IL-1α, Lipocalcin 2, MMP-9, PDGF-AA
PZP and in co-culture	Angiopoietin 1, Complement factor D, Cystatin C, EGF, ENA-78 (CXCL5), Endoglin, FGF19, HGF, SDF-1α, Thrombospondin-1, uPAR
Coculture-specific	IL-6

Figure 8f - Uncropped Blots

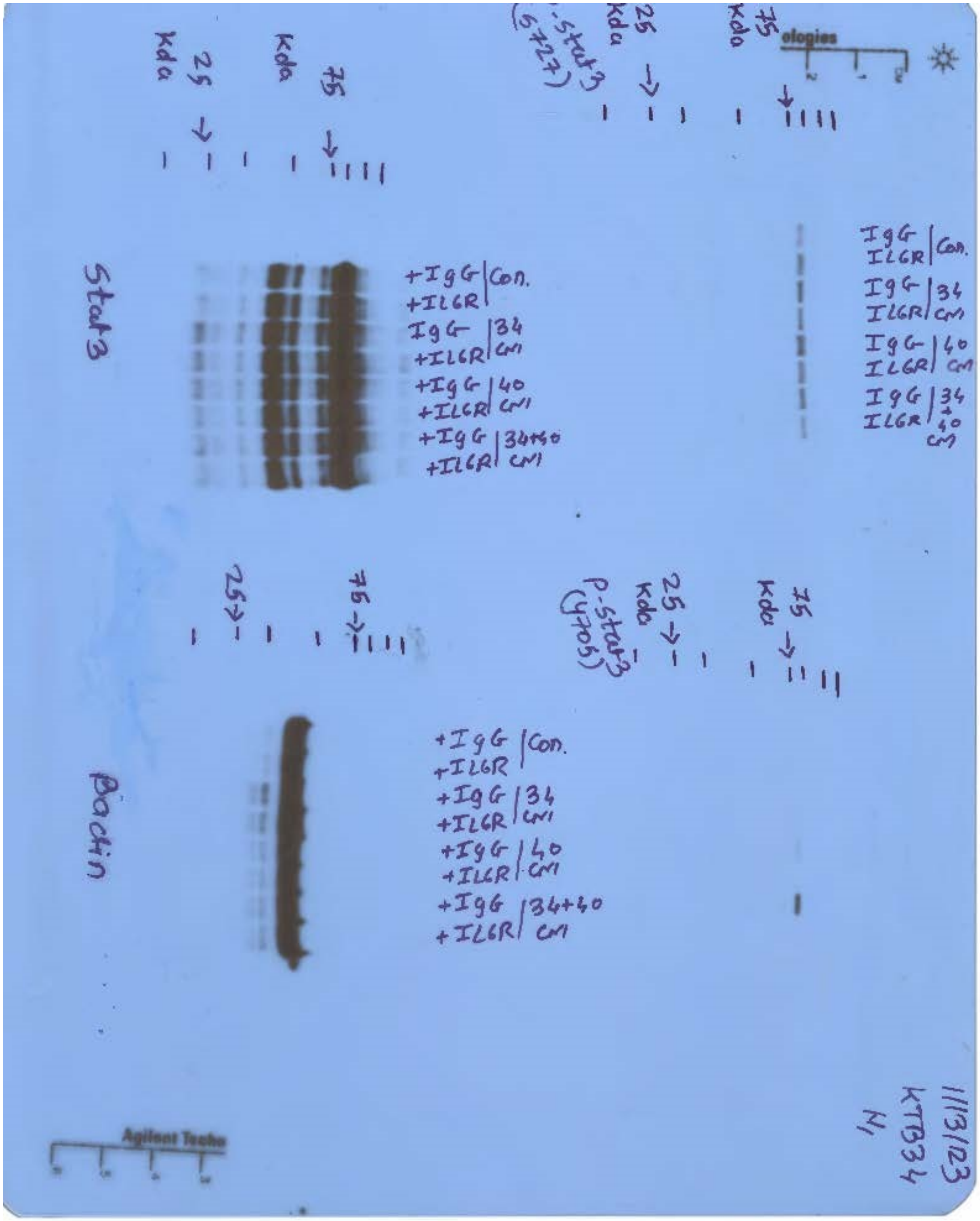
Stat3
βactin
+IgG | Con.
+IL6R
+IgG | 34 CM
+IL6R
+IgG | 40 CM
+IL6R
+IgG | 34+40 CM
+IL6R
25 Kda
75 Kda
P-Stat3 (Y705)
P-Stat3 (S727)
11/13/23
KTB34
H1

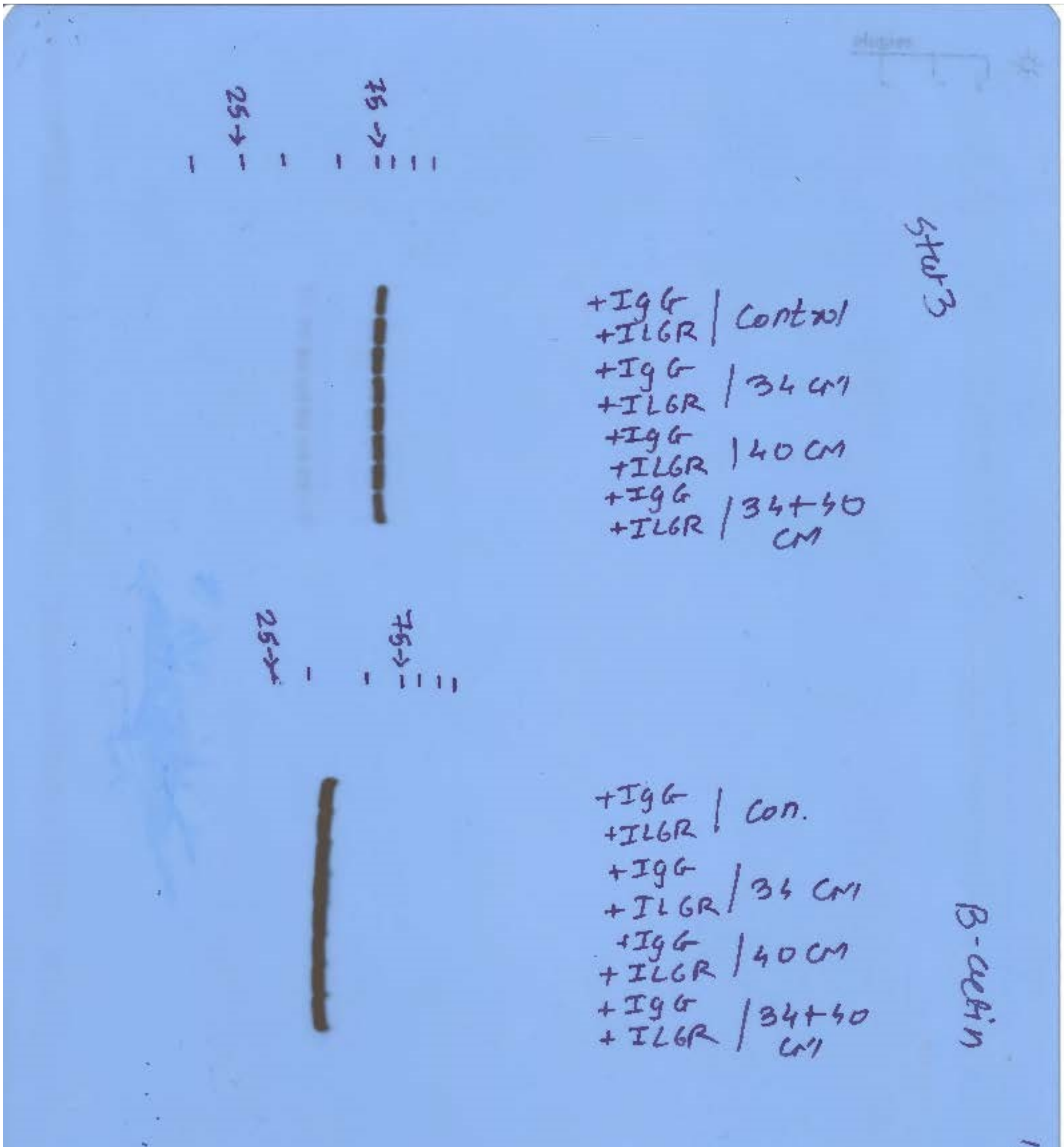
25
75
Stat3
+IgG
+ILGR | Control
+IgG
+ILGR | 34 CM
+IgG
+ILGR | 40 CM
+IgG
+ILGR | 34+40 CM
25
75
+IgG
+ILGR | Con.
+IgG
+ILGR | 34 CM
+IgG
+ILGR | 40 CM
+IgG
+ILGR | 34+40 CM
B-actin
1/13/23
KTB34

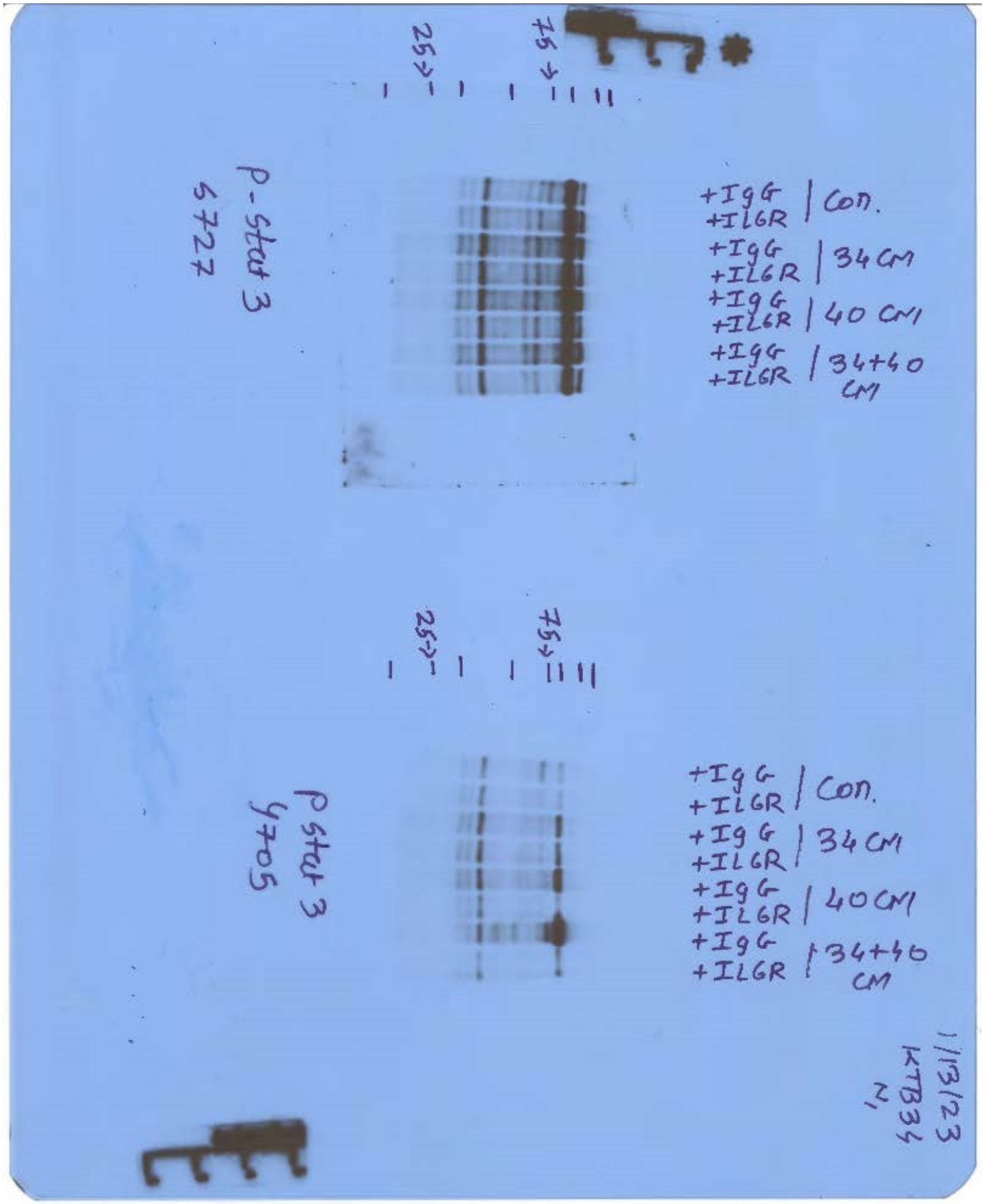
P-Stat 3
S727
+IgG
+IL6R | Con.
+IgG
+IL6R | 34 CM
+IgG
+IL6R | 40 CM
+IgG
+IL6R | 34+40 CM
P Stat 3
Y705
+IgG
+IL6R | Con.
+IgG
+IL6R | 34 CM
+IgG
+IL6R | 40 CM
+IgG
+IL6R | 34+40 CM
1/13/23
KTB34

Figure S13- Uncropped Blots

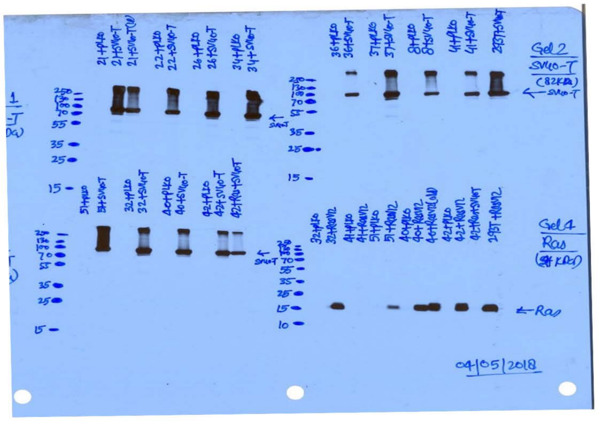

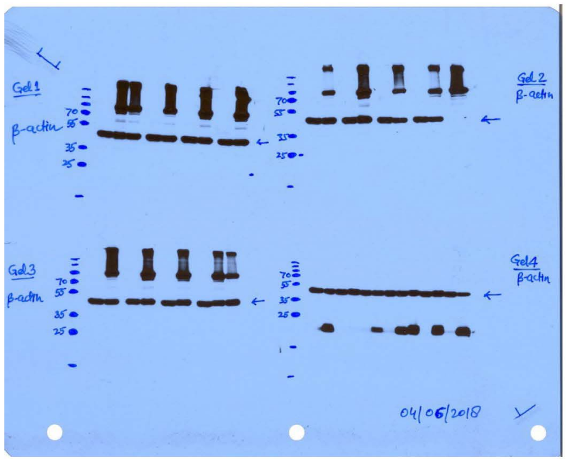

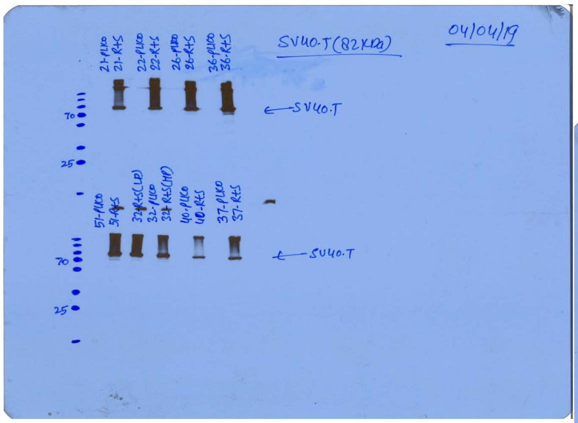

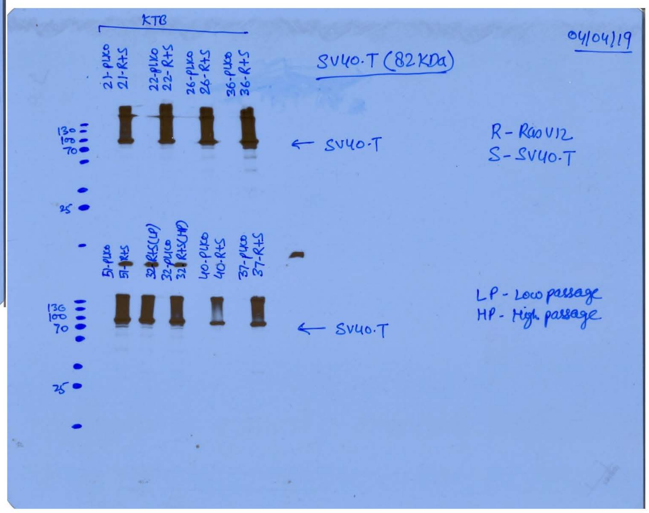

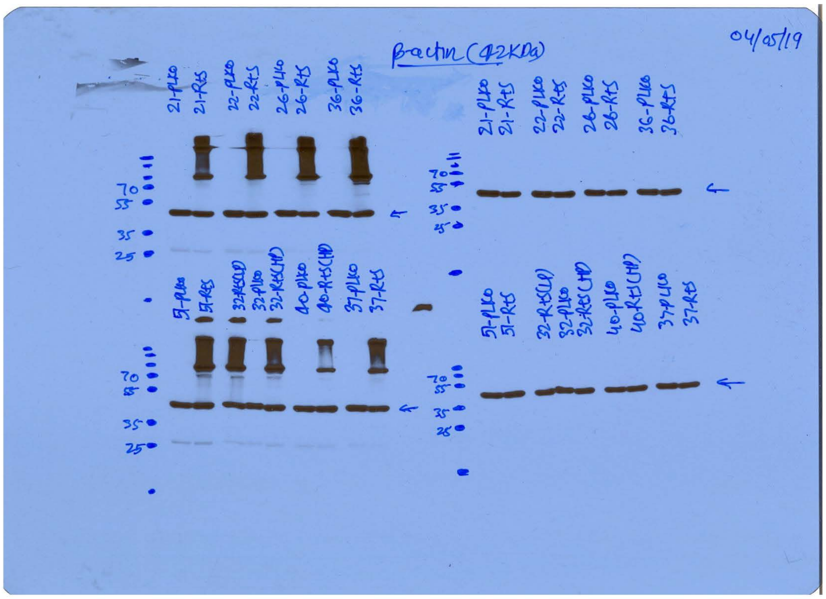
β-actin (42KDa)
04/05/19
21-PLKO
21-RES
22-PLKO
22-RES
26-PLKO
26-RES
36-PLKO
36-RES

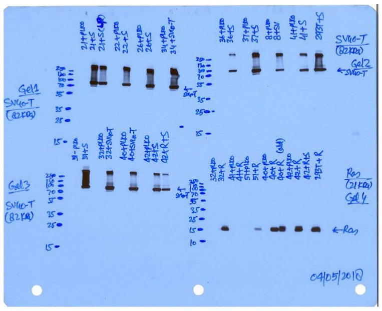

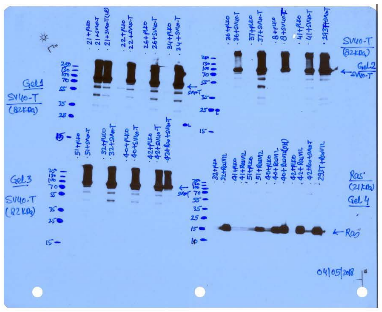

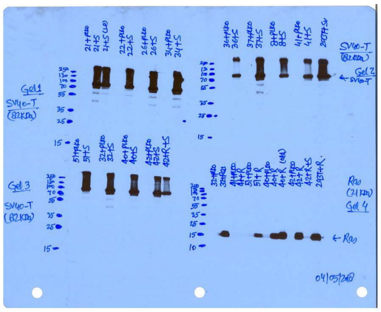

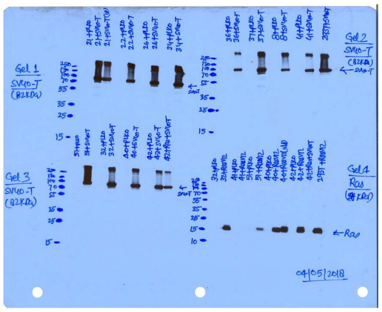

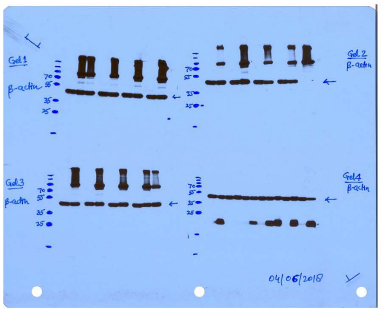

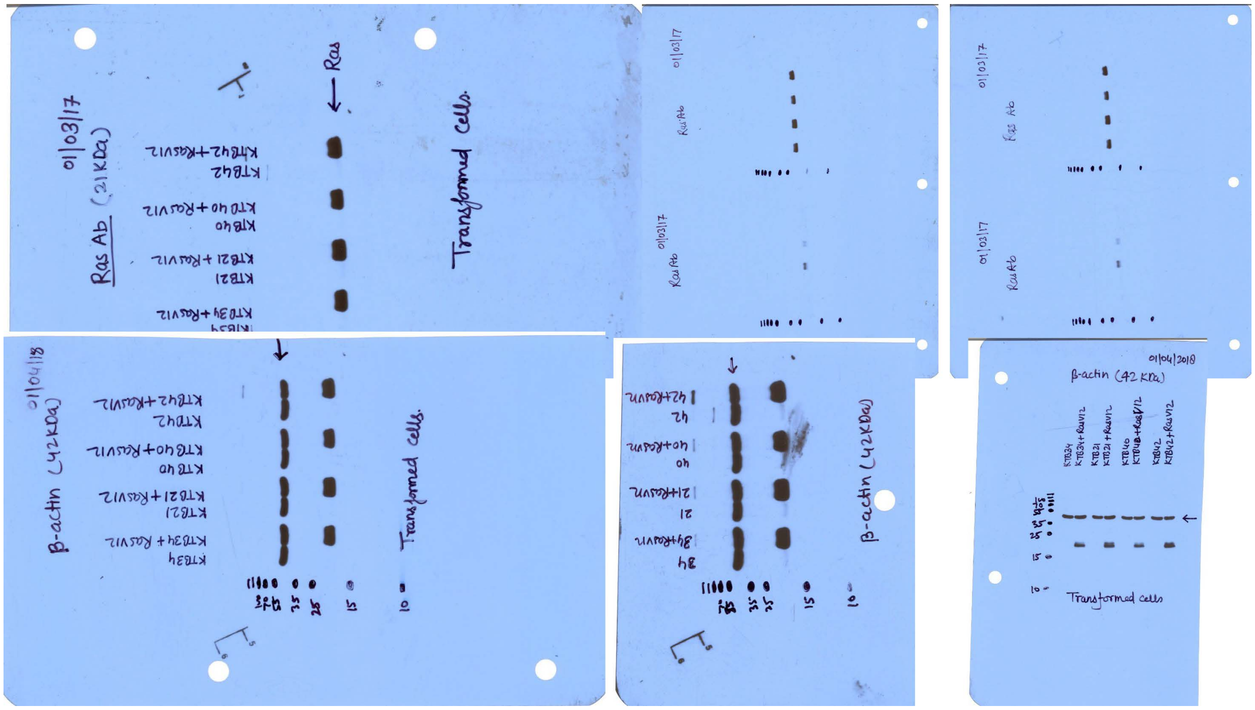

Ras Ab (21KDa)
01/03/17
Transformed cells.
β-actin (42KDa)
01/04/18
β-actin (42 KDa)
01/04/2018
Transformed cells

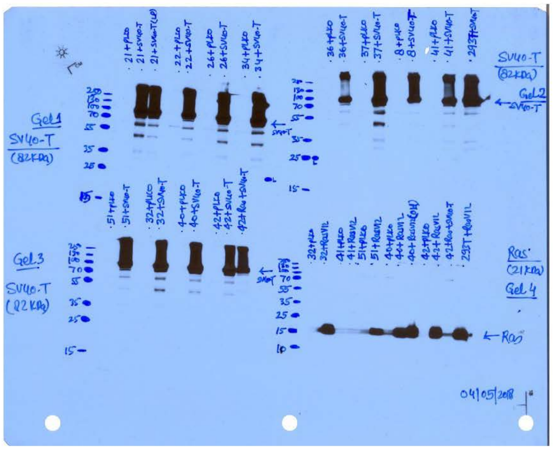

Gel1
SV40-T (82KDa)
Gel2
Gel3
SV40-T (82KDa)
Ras (21KDa)
Gel 4
04/05/2018

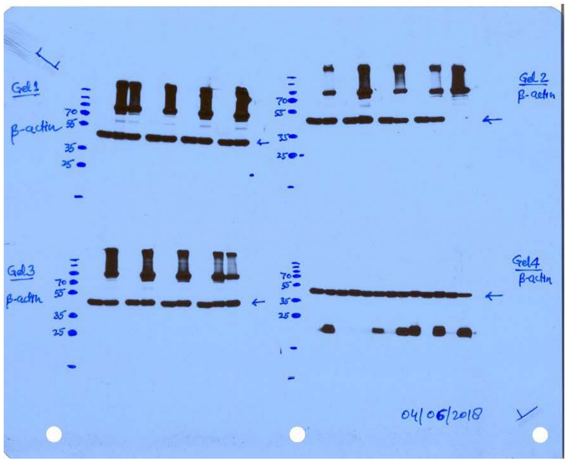

Gel1
β-actin
Gel 2
β-actin
Gel3
β-actin
Gel4
β-actin
04/06/2018

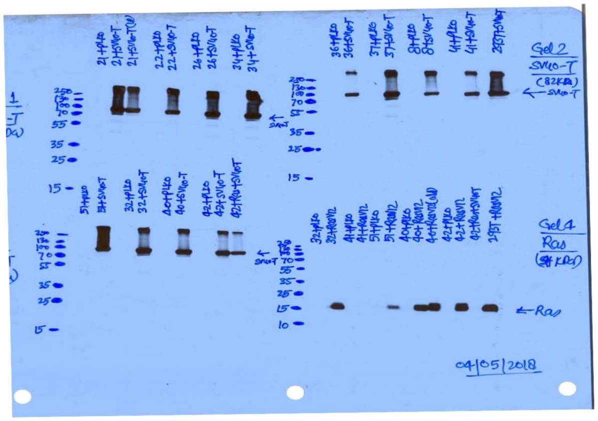
Gel 2
SV40-T
(82KDa)
Gel 4
Ras
04/05/2018

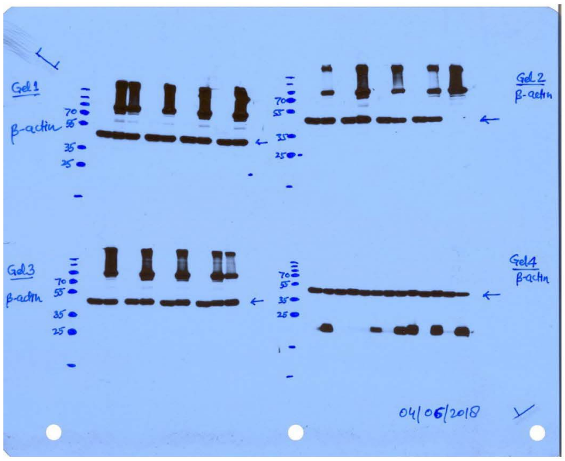
Gel 1
β-actin
Gel 2
β-actin
Gel 3
β-actin
Gel 4
β-actin
04/06/2018

References:

1. Kumar B*, et al.* Normal breast-derived epithelial cells with luminal and intrinsic subtype-enriched gene expression document inter-individual differences in their differentiation cascade. *Cancer Res* **78**, 5107-5123 (2018).
