# Supplementary material for: Stromal heterogeneity may explain increased incidence of metaplastic breast cancer in women of African descent
Source: Nat Commun. 2023 Sep 14;14:5683. doi: 10.1038/s41467-023-41473-6 (PMC10502140; doi:10.1038/s41467-023-41473-6)
Supplement: Supplementary file 3 — Reporting Summary [file 41467_2023_41473_MOESM3_ESM.pdf]

nature portfolio

Corresponding author(s): Harikrishna Nakshatri

Last updated by author(s): Jul 19, 2023

Reporting Summary

Nature Portfolio wishes to improve the reproducibility of the work that we publish. This form provides structure for consistency and transparency in reporting. For further information on Nature Portfolio policies, see our Editorial Policies and the Editorial Policy Checklist.

Statistics

For all statistical analyses, confirm that the following items are present in the figure legend, table legend, main text, or Methods section.

n/a	Confirmed	
☐	☒	The exact sample size (n) for each experimental group/condition, given as a discrete number and unit of measurement
☐	☒	A statement on whether measurements were taken from distinct samples or whether the same sample was measured repeatedly
☐	☒	The statistical test(s) used AND whether they are one- or two-sided<br>*Only common tests should be described solely by name; describe more complex techniques in the Methods section.*
☐	☒	A description of all covariates tested
☐	☒	A description of any assumptions or corrections, such as tests of normality and adjustment for multiple comparisons
☐	☒	A full description of the statistical parameters including central tendency (e.g. means) or other basic estimates (e.g. regression coefficient) AND variation (e.g. standard deviation) or associated estimates of uncertainty (e.g. confidence intervals)
☐	☒	For null hypothesis testing, the test statistic (e.g. F, t, r) with confidence intervals, effect sizes, degrees of freedom and P value noted<br>*Give P values as exact values whenever suitable.*
☒	☐	For Bayesian analysis, information on the choice of priors and Markov chain Monte Carlo settings
☒	☐	For hierarchical and complex designs, identification of the appropriate level for tests and full reporting of outcomes
☒	☐	Estimates of effect sizes (e.g. Cohen's d, Pearson's r), indicating how they were calculated

Our web collection on statistics for biologists contains articles on many of the points above.

Software and code

Policy information about availability of computer code

Data collection	Flow cytometry (BD LSR II), Cell sorting (BD FACSAria and SORPAria), Step One Plus Real Time PCR system.
Data analysis	FlowJo v10.6.2,Prism software program (version 9.0), ScanScope CS (Version 102.0.7.5)

For manuscripts utilizing custom algorithms or software that are central to the research but not yet described in published literature, software must be made available to editors and reviewers. We strongly encourage code deposition in a community repository (e.g. GitHub). See the Nature Portfolio guidelines for submitting code & software for further information.

Data

Policy information about availability of data

All manuscripts must include a data availability statement. This statement should provide the following information, where applicable:

- Accession codes, unique identifiers, or web links for publicly available datasets
- A description of any restrictions on data availability
- For clinical datasets or third party data, please ensure that the statement adheres to our policy

The authors declare that the data supporting the finding of this study are available in the paper, supplementary information, source data. This manuscript does not report any high throughput data. All reagents created as part of the manuscript will be distributed upon meeting requirements of the Indiana University.

Human research participants

Policy information about studies involving human research participants and Sex and Gender in Research.

Reporting on sex and gender	Human Female breasts were considered for study.
Population characteristics	Healthy African American, European, and Hispanic/Latina ancestry women aged between 18-93.
Recruitment	No self-selection biased involved.
Ethics oversight	Normal breast tissues of healthy African American, European, and Hispanic women were donated to the Komen Tissue Bank (KTB) after informed written consent from subjects. All experiments were carried out in accordance with the approved guidelines of the Indiana University Institutional Review Board. International Ethical Guidelines for Biomedical Research Involving Human Subjects were followed.

Note that full information on the approval of the study protocol must also be provided in the manuscript.

Field-specific reporting

Please select the one below that is the best fit for your research. If you are not sure, read the appropriate sections before making your selection.

☒ Life sciences ☐ Behavioural & social sciences ☐ Ecological, evolutionary & environmental sciences

For a reference copy of the document with all sections, see nature.com/documents/nr-reporting-summary-flat.pdf

Life sciences study design

All studies must disclose on these points even when the disclosure is negative.

Sample size	Healthy breast tissue from 49 women of African, 154 of European and 46 of Hispanic/Latinas ancestry were used in the TMA analysis. The TMA also contained matched normal adjacent to tumor and tumor tissue of approximately 50 donors. Either three or more than three samples have included in the experiments. All the available samples were included in the study without any exclusion.
Data exclusions	All the samples were included in the analysis with No exclusion.
Replication	All attempts at replication were successful.
Randomization	For IHC, Genetic ancestry analysis and statistical analysis researchers were blinded for the duration of experiment. The same number of cells were injected per experiment per animal and all animals were housed under same condition.
Blinding	Investigators were blinded to group allocation during data collection and /or analysis.

Reporting for specific materials, systems and methods

We require information from authors about some types of materials, experimental systems and methods used in many studies. Here, indicate whether each material, system or method listed is relevant to your study. If you are not sure if a list item applies to your research, read the appropriate section before selecting a response.

Materials & experimental systems

n/a	Involved in the study	
☐	☒	Antibodies
☐	☒	Eukaryotic cell lines
☒	☐	Palaeontology and archaeology
☐	☒	Animals and other organisms
☒	☐	Clinical data
☒	☐	Dual use research of concern

Methods

n/a	Involved in the study	
☒	☐	ChIP-seq
☐	☒	Flow cytometry
☒	☐	MRI-based neuroimaging

Antibodies

Antibodies used	Antibodies were used for flow cytometry and included antibodies PROCR (CD201)-PE (130-105-256, Miltenyi Biotech Inc., dilution 2:200), EpCAM-APC (130-091-254, Miltenyi Biotech Inc., dilution 2:200) EpCAM-PE (130-091-253, Miltenyi Biotech Inc., dilution

2:200), PDGFRα-PE (#323506, Biolegend, dilution 5:200), CD26-APC (#563670, BD Biosciences, dilution 5:200), CD105-PE (#12-1057-42, Invitrogen, dilution 5:200), CD73-PE (561014, BD Pharmingen, dilution 5:200), CD90-APC (559869, BD Pharmingen, dilution 5:200), CD44-APC (559942, BD Pharmingen, dilution 5:200), CD24-PE (555428, BD Pharmingen, dilution 5:200), CD10-APC (#340922, BD Biosciences, dilution 5:200), CD10-PE (#340920, BD Biosciences, dilution 5:200), CD49f-APC (FAB13501A, R&D Systems, dilution 5:200), CD44-FITC (555478, BD Biosciences, dilution 5:200), CD24-FITC (#555427, BD Biosciences, dilution 5:200), CD298-FITC (130-101-291, Miltenyi Biotech Inc., dilution 5:200), ALDEFLUOR (01700; STEMCELL Technologies, dilution 5:200), FITC (555573, BD Pharmingen, dilution 2.5:200), PE (555749, BD Pharmingen, dilution 2.5:200), and APC (555576, BD Pharmingen dilution 2.5:200). For IL-6 signaling neutralization studies, anti-IL-6R antibody (MAB227, R&D Systems, dilution 2µg/ml) was used. For western blotting antibodies used included rabbit anti-Ras (#3965, Cell Signaling Technologies), anti-pSTAT3 Y705 (#9145S, Cell Signaling Technologies, dilution 1:1000), anti-pSTAT3 S727 (#9134D, Cell Signaling Technologies, dilution 1:1000), anti-STAT3 (#4904S, Cell Signaling Technologies, dilution 1:1000), mouse anti-SV40-T antigens (MABF121, EMD Millipore, dilution 1:1000), and mouse anti-β-actin (A5441, Sigma-Aldrich, 1:5000). Anti-mouse (#7076, Cell Signaling technologies, dilution 1:10000), and anti-rabbit (#7074, Cell Signaling technologies, dilution 1:10000)

Validation

Pre-validated antibodies were purchases from well recognized vendors. Validation of each primary antibodies for the species, application, relevant citations and antibody profiles are available in online databases of manufacturer's website.

Eukaryotic cell lines

Policy information about cell lines and Sex and Gender in Research

Cell line source(s)	human breast
Authentication	Cell lines were authenticated using human marker by flow cytometry.
Mycoplasma contamination	Cell lines in the laboratory are tested negative for Mycoplasma contamination.
Commonly misidentified lines (See ICLAC register)	No commonly misidentified cell lines were used in the study.

Animals and other research organisms

Policy information about studies involving animals; ARRIVE guidelines recommended for reporting animal research, and Sex and Gender in Research

Laboratory animals	NSG (NOD/SCID/IL2Rgnull), female mice , age 5 to 6 week old were used in the study. All the animals were maintained in sterile cages with constant ambient temperature and humidity with 12 hr light/dark cycle.
Wild animals	No wild animals were used in the study.
Reporting on sex	Female mice were used in the study.
Field-collected samples	No field collected samples were used in the study.
Ethics oversight	The Indiana University Animal Care and Use Committee approved the use of animals in this study and all procedures were performed as per NIH guidelines.

Note that full information on the approval of the study protocol must also be provided in the manuscript.

Flow Cytometry

Plots

Confirm that:

☒ The axis labels state the marker and fluorochrome used (e.g. CD4-FITC).

☒ The axis scales are clearly visible. Include numbers along axes only for bottom left plot of group (a 'group' is an analysis of identical markers).

☒ All plots are contour plots with outliers or pseudocolor plots.

☒ A numerical value for number of cells or percentage (with statistics) is provided.

Methodology

Sample preparation	Freshly collected breast tissues or cryopreserved tissues were minced and incubated with collagenase/hyaluronidase mixture (300µl in 2.7ml of media, #07919, Stem Cell Technologies) plus ROCK inhibitor (5µM, #ALX-270-33-005, Enzo Life Sciences) for two hours at 37°C. The dissociated cells were filtered through sterile 70-micron filters, pelleted, washed in PBS, and plated on pre-coated conditioned medium (CM) from the 804G cells.
Instrument	BD LSR II, BD FACSAria Fusion and SORPAria

Software	FlowJo v10.6.2
Cell population abundance	Cells were gated as described below. Flow panels were optimized using positive and negative markers. PROCR+, ZEB1+, PDGFRα+ (PZP cells) along with Epithelial cells were characterized with various markers. 10000 cells/samples were analyzed in every experiment.
Gating strategy	FSC and SSC were used to ensure that only live cells were considered in the analysis. Gating was done using appropriate FITC, PE and APC isotype control antibodies for positive and negative cell populations.

☒ Tick this box to confirm that a figure exemplifying the gating strategy is provided in the Supplementary Information.
